# Microbial and metabolic features associated with outcome of infliximab therapy in pediatric Crohn’s disease

**DOI:** 10.1080/19490976.2020.1865708

**Published:** 2021-01-11

**Authors:** Yizhong Wang, Xuefeng Gao, Xinyue Zhang, Fangfei Xiao, Hui Hu, Xiaolu Li, Fang Dong, Mingming Sun, Yongmei Xiao, Ting Ge, Dan Li, Guangjun Yu, Zhanju Liu, Ting Zhang

**Affiliations:** a Department of Gastroenterology, Hepatology and Nutrition, Shanghai Children’s Hospital, Shanghai Jiao Tong University, Shanghai, China; b Institue of Pediatric Infection, Immunity and Critical Care Medicine, Shanghai Children’s Hospital, Shanghai Jiao Tong University School of Medicine, Shanghai, China; c Hematology-Oncology, International Cancer Center, Shenzhen University General Hospital, Shenzhen University Health Science Center, Shenzhen, China; d Department of Gastroenterology, The Shanghai Tenth People’s Hospital of Tongji University, Shanghai, China

**Keywords:** Children, Crohn’s disease, gut microbiome, infliximab, metabolome

## Abstract

Gut microbial dysbiosis and altered metabonomics have been implicated in the pathogenesis of Crohn’s disease (CD). The aim of our study was to characterize the gut microbiome structure and metabolic activities in pediatric CD patients with different clinical outcomes after infliximab (IFX) therapy. Fecal samples were collected from 20 healthy children and 29 newly diagnosed pediatric CD patients. 16S rRNA/ITS2 gene sequencing and targeted metabolomics analysis were applied to profile the gut bacterial microbiome, mycobiome, and metabolome, respectively. Pediatric CD patients exhibited lower relative abundances of short-chain fatty acids (SCFAs)-producing bacteria including *Faecalibacterium, Clostridium* clusters IV and XIVb, *Roseburia*, and *Ruminococcus*, which were correlated with reduced fecal levels of SCFAs. Decreased unconjugated bile acids (BAs) pool size and a lower unconjugated/conjugated BAs ratio were associated with reduced relative abundances of *Bifidobacterium* and *Clostridium* clusters IV and XIVb which contain bile salt hydrolases (BSH) genes. IFX treatment enriched the BSH-producing bacteria in CD subjects, which may explain a decreased level of conjugated BAs and an increase in unconjugated BAs as well as the unconjugated/conjugated BAs ratio. Furthermore, a sustained response (SR) of IFX therapy was associated with higher abundances of *Methylobacterium, Sphingomonas, Staphylococcus*, and *Streptococcus*, and higher fecal concentrations of amino acids, including L-aspartic acid, linoleic acid, and L-lactic acid at baseline. Our study suggests that the effects of IFX might be partially mediated by enriching bacteria taxa that producing SCFAs and BSH thereby inhibiting inflammation and restoring the BA metabolism. Some fecal bacteria and metabolites may be predictive of outcomes of IFX therapy for pediatric CD patients.

## Introduction

Crohn’s disease (CD) is a major form of inflammatory bowel diseases (IBD) characterized by chronic transmural inflammation, mucosal aphthous ulcers, and noncaseating granulomas in the gastrointestinal (GI) tract.^1^ The incidence rate of CD in the pediatric population is steadily increased in past two decades, particularly in the newly developed countries.^2,3^ Children with CD usually suffered episodes of disease remission and relapse that significantly impair their growth and development. The exact etiology and pathogenesis of CD are still unclear, but emerging evidence indicates that CD is strongly associated with disturbances to the gut microbiota.^4–7^

Currently, there are several studies^8–12^ reported the alterations of gut bacterial microbiota in pediatric IBD patients. The numbers of *Coriobacteriaceae* and *Lachnospiraceae*, and the diversity of *Ruminococcaceae* and *Bifidobacterial* population were lower in pediatric IBD patients as compared with the healthy children.^9^ It was reported that the abundances of bacteria within families *Enterobacteriaceae, Fusobacteriaceae, Pasteurellacaea*, and *Veillonellaceae* were higher, and abundances in orders Bacteroidales, Clostridiales, and Erysipelotrichales were lower in newly diagnosed pediatric CD patients than the healthy children.^5^ Our previous study^12^ showed that the fecal microbiota of pediatric CD patients was represented by a lower biodiversity, a gain in *Enterococcus*, and a significant loss in multiple short-chain fatty acids (SCFAs)-producing bacteria. Beyond bacteria, other microorganisms presented in gut microbiota, such as fungi, have been suggested to play a role in IBD pathogenesis. The dominating fungal microbiota was species with Basidiomycota in de-novo pediatric IBD patients, while Ascomycota was predominated in healthy subjects.^13^ Chehoud et al. reported a lower diversity of mycobiota and an increasing abundance of *Candida* spp. in pediatric IBD patients.^14^ Compared to the healthy children, *Psathyrellaceae*, *Cortinariaceae*, *Psathyrella*, and *Gymnopilus* were significantly enriched, whereas Monilinia was significantly depleted in CD children.^7^ However, the prior results on this topic have been inconsistent.

The gut microbiota interacts with the host mainly through signals triggered by microbial metabolites. Changes in the gut microbiota correspond with altered microbial metabolic functions have been implicated in the pathogenesis of IBD.^15^ Metabolomic profiling of biofluids in IBD patients may help to find novel biomarkers for disease diagnosis and monitoring, and reveal the role of metabolic alterations in disease development and progress. Alterations in a number of metabolites in patients with IBD were confirmed by metabolomics studies, such as fecal bile acids (BAs) and SCFAs.^15,16^ Metabolic profiling of serum samples revealed that numbers of chemically annotated metabolites belong to phospholipids were downregulated in CD children.^17^ Kolho et al. showed that serum and fecal metabolite profiles in newly diagnosed pediatric IBD patients were different from healthy children, neopterin was the top metabolite in the discriminant analysis with a high level in serum of CD.^18^ Nonetheless, the microbial origins of CD-associated metabolites and the mechanisms underlying gut microbiota-mediated changes in CD metabolome have not been fully investigated.

A prolonged treatment with anti-tumor necrosis factor (TNF) agents, such as infliximab (IFX), is frequently advised for moderate to severe CD patients. Despite high primary response rates of 70–90%, the long-term outcome of CD patients treated with IFX is still suboptimal.^19^ Many CD patients with an initial clinical response have recurrent symptoms during IFX maintenance therapy, and nearly 40% of them lost the clinical responses eventually.^20^ Some biomarkers for predicting response to anti-TNF treatment have been identifed, including age, body mass index (BMI), duration of disease, fecal calprotectin, and serological antibodies.^21^ Recent studies have suggested that assessing the gut microbial community and metabolomics may provide new insights into anti-TNF treatment optimization.^12,22–26^ It was reported that increased *Bifidobacterium, Collinsella, Lachnospira, Lachnospiraceae, Roseburia, Eggerthella* taxa and reduced *Phascolarctobacterium* were associated with treatment success of anti-TNF therapy in CD patients.^27^ *F. prausnitzii* represented a predictive factor for recurrence after IFX discontinuation in CD.^23^ We previously showed that a sustained response (SR) of IFX therapy in CD children was positively associated with an expansion of SCFA-producing bacteria, especially the *Blautia, Faecalibacterium, Lachnospira*, and *Roseburia*.^12^ Metabolite profiling of serum and urine identified histidine and cysteine as biomarkers of response to anti-TNF therapy in adult CD.^26^ Furthermore, fecal levels of butyrate and substrates involved in butyrate synthesis were significantly associated with clinical remission of CD patients following anti-TNF therapy.^24^

In the current study, we aim to investigate the characteristics of fecal microbiome (bacteria and fungi) and metabolome, and attempt to explore their relationships and functional roles in clincial response of IFX therapy in a group of pediatric CD patients.

## Results

### Responses of pediatric CD to IFX therapy

Demographic and clinical characteristics of the 29 pediatric CD patients included in this study were shown in (Table 1). The median age of the 29 CD patients was 13 y (interquartile range (IQR), 11–14 y), and 21 were boys. The majority of patients (25/29, 86.2%) having ilecolonic disease (L3). These CD patients had a median baseline PCDAI score of 32.5 (IQR, 20.0–37.5) and with a median CRP of 41 mg/L (IQR, 15–56 mg/L). During the study period, 18 patients received 3–6 times of IFX therapy. A total of 11 patients achieved SR, defined as (1) a PCDAI ≤10 at each follow-up time point, or (2) a ≥10-point reduction in the PCDAI from baseline to after the third IFX infusion and PCDAI ≤10 after the sixth IFX infusion. Furthermore, significant decreases in WBC, ESR, PLT, and CRP values were observed in SR patients after IFX therapy (Table S1). Among seven patients with non-sustained response (NSR, PCDAI >10 after sixth IFX therapy), two patients (CD06, CD12) had early clinical response after third IFX therapy; however, they lost response in the subsequent IFX maintenance therapy.

**Table 1.** Baseline characteristics of pediatric Crohn’s disease (CD) patients and health subjects

Characteristic	Healthy subjects (*n* = 20)	All CD (*n* = 29)	CD treated with IFX (*n* = 18)	*P*^#^
Age, y, median (IQR)	12 (11, 13)	13 (11, 14)	12 (11, 14)	.859
Male, *n* (%)	15 (75.0)	21 (72.4)	12 (63.2)	.846
Disease location (ParisL)				
L2, *n* (%)		2 (6.9)	2 (11.1)	.619
L3, *n* (%)		25 (86.2)	14 (77.8)	.46
L4, *n* (%)		2 (6.9)	2 (11.1)	.619
Blood tests				
WBC (×10^9^/L), median (IQR)		12.0 (8.0, 17.8)	11.9 (8.3, 17.6)	.743
ESR (mm/h), median (IQR)		64 (42, 104)	64 (35, 106)	.852
PLT (×10^9^/L), median (IQR)		443 (326, 515)	464 (434, 527)	.381
CRP (mg/L), median (IQR)		41 (15, 56)	41 (18, 58)	.848
PCDAI, median (IQR)		32.5 (20.0, 37.5)	33.8 (25.6, 39.4)	.475
Outcome of IFX therapy				
SR, *n* (%)			11 (61.1)	
NSR, *n* (%)			7 (38.9)	

^#^The data were compared by the nonparametric Mann–Whitney test (two groups) or Kruskal–Wallis *H* test (multiple groups).

CD, Crohn’s disease; IFX, infliximab; SR, sustained response; NSR, non-sustained response; WBC, white blood cells; ESR, erythrocyte sedimentation rate; PLT, platelets; CRP, C-reactive protein; PCDAI, Pediatric Crohn’s Disease Activity Index.

### Pediatric CD is linked to specific fecal microbiome and mycobiome fingerprints

**Figure 1.**
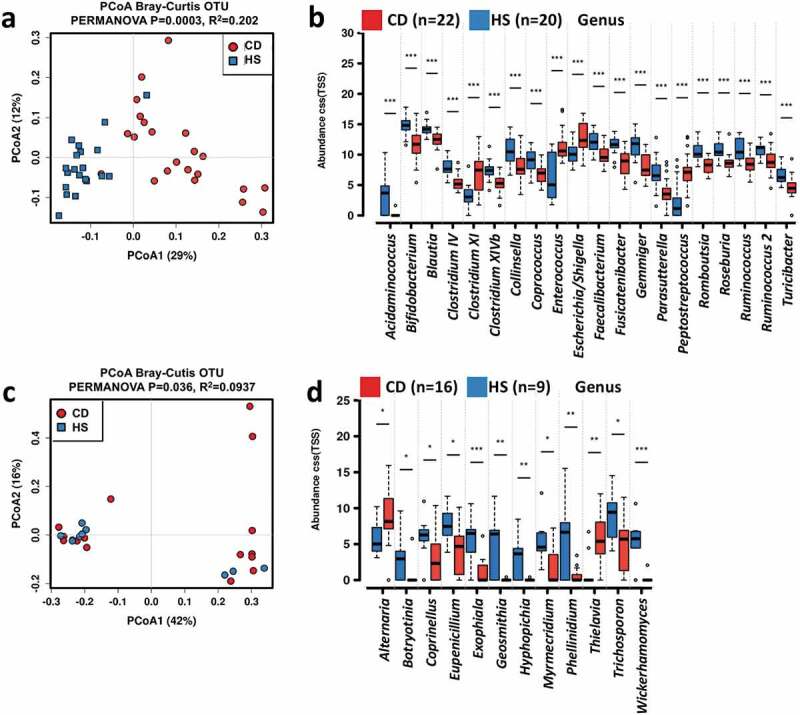
Altered fecal microbiome biodiversity and composition in the pediatric CD patients. Beta diversity of bacterial (a) and fungal (c) microbiome. PCoA of Bray–Curtis distance with each sample colored according to the phenotype. PC1 and PC2 represent the top two principal coordinates that captured most of the diversity. The fraction of diversity captured by the coordinate is given as a percentage. Groups were compared using PERMANOVA method. Boxplots showing the significantly different bacterial genera (b) and fungal genera (d), and their relative abundances (in log2(CSS)) in HS and CD patients before IFX treatment. Significance is determined by using Wilcoxon rank-sum test, with *P < .05, **P < .01, ***P < .001, and #FDR<0.05. CD, Crohn’s disease; HS, healthy subjects; PCoA, principal coordinate analysis

Regarding the 16S rRNA data, the read counts per sample are 186, 189 in average, ranging from 36,481 to 525, 028. After sequence processing and filtering, we obtained a total of 1,628,685 16S rRNA gene sequences spanning a total of 2,872 bacterial OTUs. No significant difference in the alpha diversity of bacterial communities was identified between CD and HS, except Chao 1 (Figure S1a). The bacterial microbiota was dominated by phyla Actinobacteria, Bacteroidetes, Firmicutes, and Proteobacteria in both pediatric CD patients and HS (Figure S1b). The top three most abundant genera in HS were *Bacteroides, Bifidobacterium*, and *Blautia*, whereas potentially pathogenic genus *Escherichia/Shigella* was more prevalent in the pediatric CD patients at baseline (Figure S1c). Multidimensional scaling revealed a significant clustering of samples according to the occurrence of CD (Figure 1a). Inter-group comparisons of taxonomic profiles at the genus level revealed that fecal samples from pediatric CD patients exhibited lower relative abundances of *Acidainococcus, Bifidobacterium, Blautia, Clostridium IV, Clostridium XIVb, Faecalibacterium, Fusicatenibacter, Gemmiger, Parasutterella, Romboutsia, Roseburia, Ruminococcus*, and *Turicibacter*, and higher relative abundances of *Clostridium XI, Enterococcus, Escherichia/Shigella*, and *Peptostreptococcus* as compared to that of HS (Figure 1b).

As to the ITS data, the average counts per sample are 278,523, ranging from 6,955 to 117,618. We obtained a total of 828,553 ITS2 gene sequences spanning a total of 1,250 fungal OTUs. No statistically significant difference in the alpha diversity of fungal community was detected between samples from pediatric CD patients and HS (Figure S1d). In addition, no clear separation was observed between fungal community structure of CD and HS (Figure 1c). The fungal microbiome was dominated by phyla As comycota and Basidiomycota in both the pediatric CD patients and HS (Figure S1e), and the most commonly observed genera were *Saccharomyces, Candida, Aspergillus*, and *Gibberella* (Figure S1f). Compared to that of HS, fecal samples from the pediatric CD patients exhibited higher relative abundances of *Alternaria* and *Thielavia*, and lower relative abundances of *Botryotinia, Coprinellus, Eupenicillium, Exophiala, Geosmithia, Hyphopichia, Myrmecridium, Phellinidium, Trichosporon*, and *Wicherhamomyces* (Figure 1d).

### Dysmetabolism in the pediatric CD is characterized by reduced SCFA production and altered BA profile

**Figure 2.**
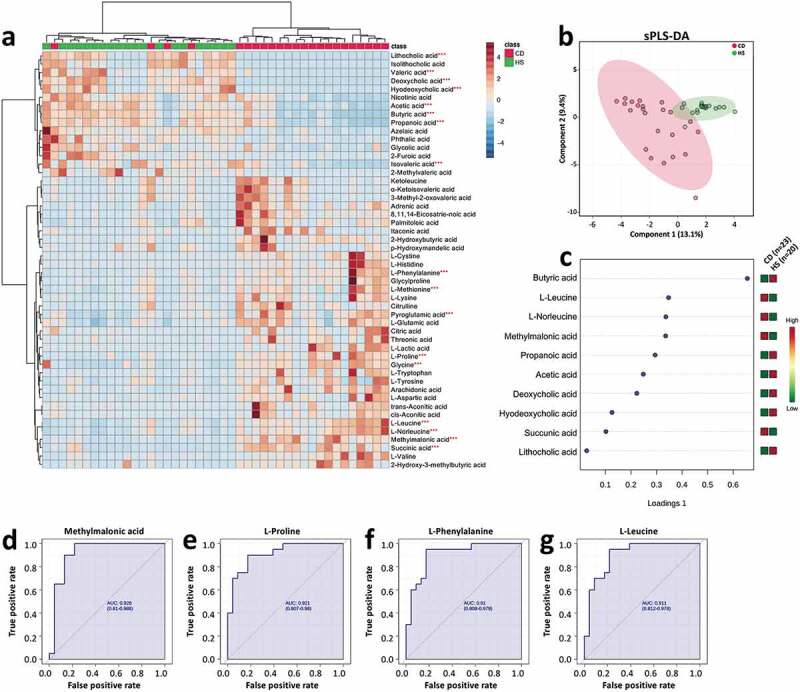
Altered fecal metabolomics in the CD patients compared with the HS. (a) Heat map showing the top 50 metabolites that different between the CD and HS, and the significance was determined by using Wilcoxon rank-sum test (***P < .001). (b) Supervised clustering of fecal metabolites using sPLS-DA. (c) The top metabolites ranked by VIP scores. (d–g) ROC analysis of potential biomarkers (AUC>0.9) for differentiating the CD patients from HS. CD, Crohn’s disease; HS, healthy subjects; sPLS-DA, sparse partial least squares discriminant analysis; VIP, variable importance in projection

A total of 215 metabolites were detected and quantified in all the fecal samples, and different distribution patterns of metabolites between CD and HS were observed (Figure 2a, b). Among the ten metabolites having the most discriminative (i.e., the highest loading values according to VIP) for classification between the two groups, CD patients showed higher fecal levels of two amino acids (L-leucine and L-norleucine) and two organic acids (methylmalonic acid, succinic acid), and lower levels of all major SCFAs (acetic acid, butyric acid, and propanol acid) and three BAs (deoxycholic acid, hyodeoxycholic acid, and lithocholic acid) (Figure 2c). Notably, the reduced levels of SCFAs in the CD patients could be well correlated with the lower abundances of multiple SCFA-producing taxa assigned to Firmicutes phylum, such as *Faecalibacterium, Clostridium* clusters IV and XIVb, *Roseburia*, and *Ruminococcus* (Figure 1b). ROC analysis showed methylmalonic acid, L-proline, L-phenylalanine, and L-leucine presenting the highest discrimination value (Figure 2d-g; AUC > 0.9).

Alterations in BAs metabolism are often seen as a consequence of changes in the gut microbial composition by affecting the bile salt biotransformation. Our data showed that total BAs were not significantly different between CD and HS (Figure S2a). However, the pediatric CD group showed a higher level of conjugated and lower level of unconjugated BAs (Figure S2b, c). Along this line, the ratio of unconjugated/conjugated BAs was also lower in the CD samples (Figure S2d). Moreover, the level of primary BAs was higher, and secondary BAs was lower in the CD children, which led to a lower secondary/primary BA ratio than that of HS (Figure S2e-g).

### Putative mechanistic associations between CD-related gut microbes and metabolites

**Figure 3.**
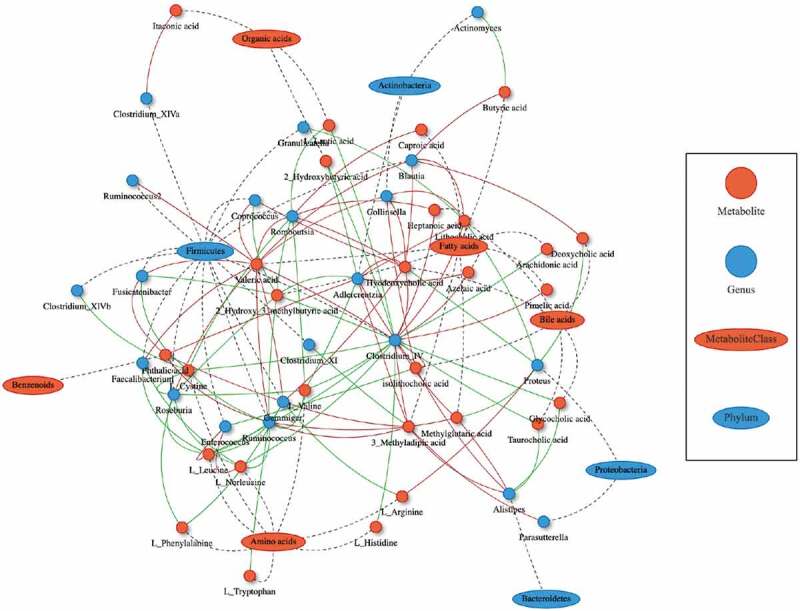
Potentially mechanistic associations between CD-linked microbes and metabolites. Network of the top 100 pairwise metabolite-microbe correlations. Metabolites and microbes are represented as red circles and blue ovals. Their positive and negative correlations are indicated using red and green color, respectively. Differential metabolites and bacteria between the CD and HS at baseline (Mann–Whitney *U* test, *P* < .01) were selected for the Spearman correlation coefficients. Coefficient values *R* ≤ −0.5 and ≥ 0.5 with *P* value < .01 were considered statistically significant, and were plotted in network. CD, Crohn’s disease; HS, healthy subjects

In order to couple the alterations of the gut microbiome and dysmetabolism, Spearman rank correlations were calculated between the fecal concentrations of metabolites, and the abundances of bacterial/fungal taxonomic groups. A total of 399 significant bacteria-metabolite correlations were identified at the genus level (Figure 3). With a cutoff value of 0.6 for Spearman correlation co-efficient, we further identified 80 strong correlations between 26 metabolites (8 amino acids, 8 fatty acids, 6 BAs, 3 organic acids, and 1 benzenoid) and 20 bacterial genera (12 belong to the Firmicutes phylum, Table S2).

Gut bacterial metabolism plays vital roles in determining the intestinal pool of bile salts. In the intestine, the primary BAs, cholic acid and chenodeoxycholic acid are converted by intestinal bacteria to form the secondary BAs, deoxycholic acid, and lithocholic acid, respectively. This transformation is processed via deconjugation that is catalyzed by bile salt hydrolases (BSH), an enzyme expressed predominantly by *Bacteroides, Bifidobacteria, Clostridium*, and *Lactobacillus*, and followed by 7α-dehydroxylase (mainly expressed by *Clostridium* and *Eubacterium*).^28^ The gut microbiome analysis revealed lower abundances of genera *Bifidobacteria*, and *Clostridium* (clusters IV and XI) in the pediatric CD patients (Figure 1b), which may explain the cause of reduced unconjugated BAs pool and lower unconjugated/conjugated BAs ratio (Figure S2c, d).

### IFX modified the gut bacterial, fungal microbiome, and metabolome

**Figure 4.**
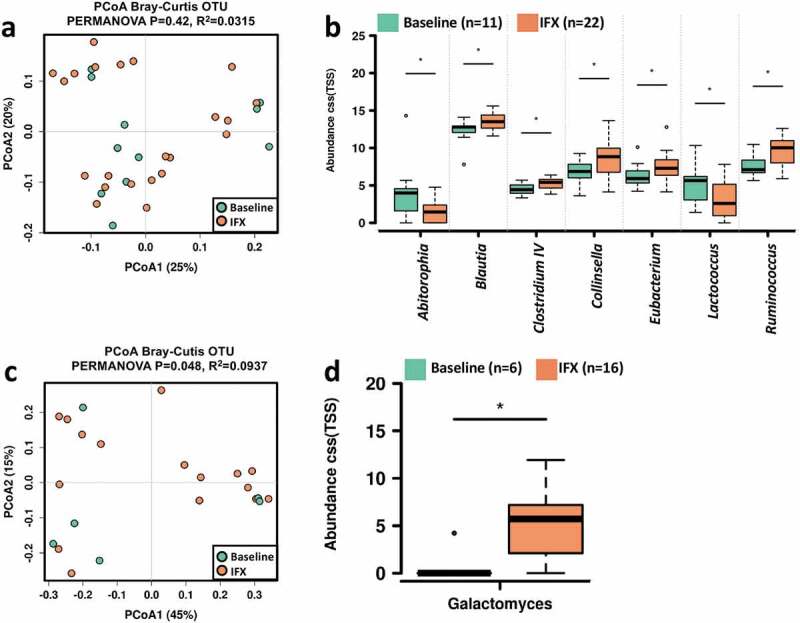
Changes in the fecal microbiome biodiversity and composition in the pediatric CD patients after IFX treatment. Beta diversity of bacterial (a) and fungal microbiome (c). PCoA of Bray–Curtis distance with each sample colored according to the phenotype. PC1 and PC2 represent the top two principal coordinates that captured most of the diversity. The fraction of diversity captured by the coordinate is given as a percentage. Groups were compared using PERMANOVA method. Boxplots showing the significantly different bacterial genera (b) and fungal genera (d) in the CD patients before and after IFX treatment. The relative abundances were shown in log2(CSS). Significance is determined by using Wilcoxon rank-sum test, with *P < .05. CD, Crohn’s disease; IFX, infliximab; NSR, non-sustained response; PCoA, principal coordinate analysis; SR, sustained response

We next looked at the variations of bacterial, fungal communities, and metabolome in feces by discriminating samples before and after IFX treatment. To avoid the effect of inter-individual differences on the interpretation of the results, only CD subjects provided both baseline and post-treatment fecal samples were included in the analysis. The bacterial community diversity was not significantly different in the CD patients before and after IFX treatment (Figure S3a). PCoA showed that the IFX-treated samples did not form a cluster of samples that distinguished from the treatment-naïve samples (Figure 4a). Comparisons of the bacterial microbiome profile revealed that the relative abundances of *Blautia, Clostridium IV, Collinsella, Eubacterium*, and *Ruminococcus* were increased, whereas *Abitorophia* and *Lactococcus* were decreased in the CD patients after IFX treatment (Wilcoxon rank-sum test *P* < .05; Figure 4b). Regarding the fecal mycobiota, no statistically significant change in alpha diversity was detected after IFX treatment (Figure S3b), and PCoA did demonstrate clustering of post-IFX samples (Figure 4c). At the genus level, an increased relative abundance of *Galactomyces* was observed after IFX therapy (Wilcoxon rank-sum test *P* < .05; Figure 4d).

By using Spearman rank correlation, we identified 21 bacterial genera with significant correlations with the severity of pediatric CD (Figure S4a). In specific, the PCDAI score and some serum indexes of inflammation, including CRP, WBC, PLT, and ESR, were positively correlated with the relative abundances of *Mycoplasma, Parasutterella, Finegoldia*, and *Haemophilus*, whereas negatively correlated with *Blautia, Eggerthella*, and *Eubacterium*. Regarding the fungal microbiota, we found that PCDAI score was negatively correlated with the relative abundances of *Galactomyces*, while positively correlated with *Rhodosporidium* and *Kabatiella* (Table S3).

**Figure 5.**
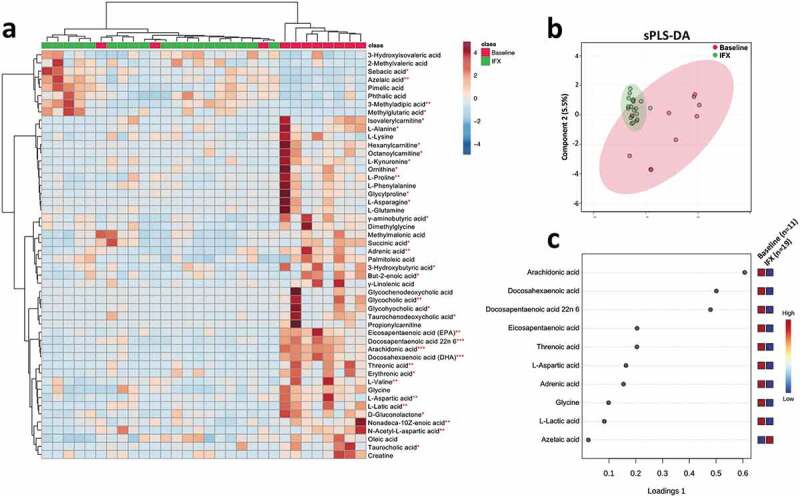
Fecal metabolomics changes in the pediatric CD patients after IFX treatment. (a) Heat map showing the top 40 metabolites that with significantly changed (*T*-test, *P* < .05) in the pediatric CD patients after IFX treatment. (b) Supervised clustering of fecal metabolites using sPLS-DA; (c) The top metabolites ranked by VIP scores. CD, Crohn’s disease; IFX, infliximab; sPLS-DA, sparse partial least squares discriminant analysis; VIP, variable importance in projection

With respect to fecal metabolome, profound metabolic changes were detected in the pediatric CD patients after IFX treatment (Figure 5a, b). We further identified ten metabolites that were most responsible for separation of the IFX-treated samples from that of baseline. In specific, the concentrations of amino acids L-aspartic acid, glycine, and organic acid, arachidonic acid, docosahexaenoic acid, docosapentaenoic acid 22n6, eicosapentaenoic acid, L-lactic acid, and threnoic acid were decreased, while azelaic acid was increased in the IFX-treated samples (Figure 5c).

In the fecal BA pool (Figure S5), the size of conjugated BAs was significantly decreased (Figure S5b), while the ratio of unconjugated/conjugated BAs ratio was significantly uplifted after IFX treatment (Figure S5d), which was in accord with an increase in the relative abundances of *Blautia* and *Collinsella* (Figure 4b) that containing BSH-producing species.^29^ Moreover, IFX treatment induced a significant increase in the ratio of secondary/primary BAs (Figure S5g), albeit an increase in the level of secondary BAs did not achieve a statistical significance (Wilcoxon rank-sum test, *P* = .078; Figure S5f). This phenomenon may be explained by an increase in 7α-dehydroxylating genera including *Clostridium IV* and *Eubacterium* (Figure 4b).

Spearman rank correlation analysis found 13 amino acids (L-alanine, L-aspartic acid, L-cystine, L-glutamic acid, L-histidine, L-kynurenine, L-leucine, L-norleucine, L-proline, L-phenylalanine, glycine, glycylproline, and *N*-acetyl-L-aspartic acid), 3 fatty acids (docosapentaenoic acid 22n6, arachidonic acid, and eicosapentaenoic acid), 1 BA (glycochenodeoxycholic acid), 2 dicarboxylic acids (methylmalonic acid and succinic acid), 4 organic acids (cis-aconitic acid, trans-aconitic acid, citric acid, and isocitric acid) were positively correlated with PCDAI and other serum indexes of inflammation (Figure S4b). In contrast, azelaic acid, butyric acid, deoxycholic acid, isovaleric acid, valeric acid, and sebacic acid were negatively correlated with the CD disease severity (Figure S4b). Moreover, the PCDAI-correlated bacteria and metabolites were also strongly correlated with each other (Figure S4c), indicating their interactions were closely associated with the severity of inflammation in the CD patients and the outcomes of IFX therapy.

### Microbial and metabolic features indicating therapeutic outcomes of IFX

**Figure 6.**
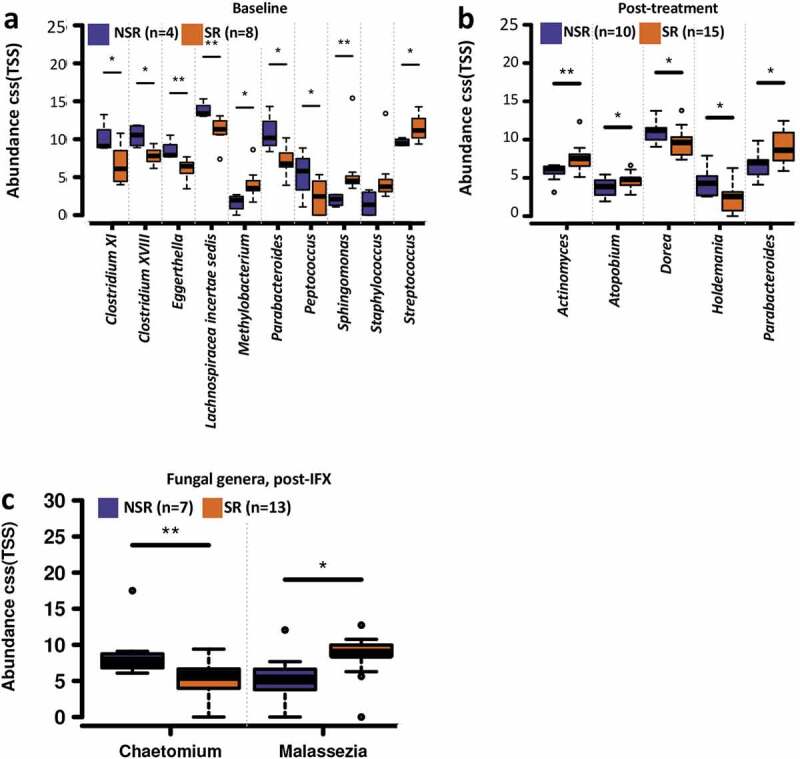
Bacterial and fungal genera correlate with outcome of the pediatric CD patients with IFX treatment. Boxplots showing the significantly different bacterial genera between the SR and NSR patients before (a) and after IFX (b) treatment, and the significantly different fungal genera between the SR and NSR patients after IFX treatment (c). The relative abundances were shown in log2(CSS). Significance is determined by using Wilcoxon rank-sum test (FDR<0.5), with *P < .05 and **P < .01. CD, Crohn’s disease; IFX, infliximab; NSR, non-sustained response; SR, sustained response

We next tried to identify specific features of the gut bacterial, fungal microbiome, and metabolome that indicative of therapeutic outcomes of IFX. The stool samples classified for the SR patients who did not immediately achieve PCDAI ≤10 (second sample for CD15, CD17, and CD19) were treated as response samples, and stool samples for NSR patients who initially responded (second stool sample for CD12) were treated as non-response in the data analysis (Table S1). No difference in the gut bacterial alpha diversities between SR and NSR patients was observed before (Figure S6a) and after (Figure S6b) IFX treatment. Comparisons of taxonomic profiles of samples from the CD patients before IFX treatment showed that the relative abundances of several bacterial genera of the NSR subjects were significantly higher than that of SR, including *Clostridium XI, Clostridium XVIII, Eggerthella, Lachnospiracea incertae sedis, Parabacteroides*, and *Peptococcus*, while the SR patients had higher abundances of *Methylobacterium, Sphingomonas, Staphylococcus*, and *Streptococcus* (Wilcoxon rank-sum test, *P* < .05; Figure 6a). After IFX therapy, the genera *Actinomyces, Atopobium*, and *Parabacteroides* were more abundant in the SR patients, whereas *Dorea* and *Holdemania* were found to be more abundant in the NSR patients (Wilcoxon rank-sum test, *P* < .05; Figure 6b).

No significant difference in the gut fungal alpha diversity was identified between the SR and NSR patients before (Figure S6c) and after (Figure S6d) IFX treatment. Compositionally, the NSR subjects showed a higher relative abundance of *Chaetomium* and a lower abundance of *Malassezia* after IFX treatment (Figure 6c).

**Figure 7..**
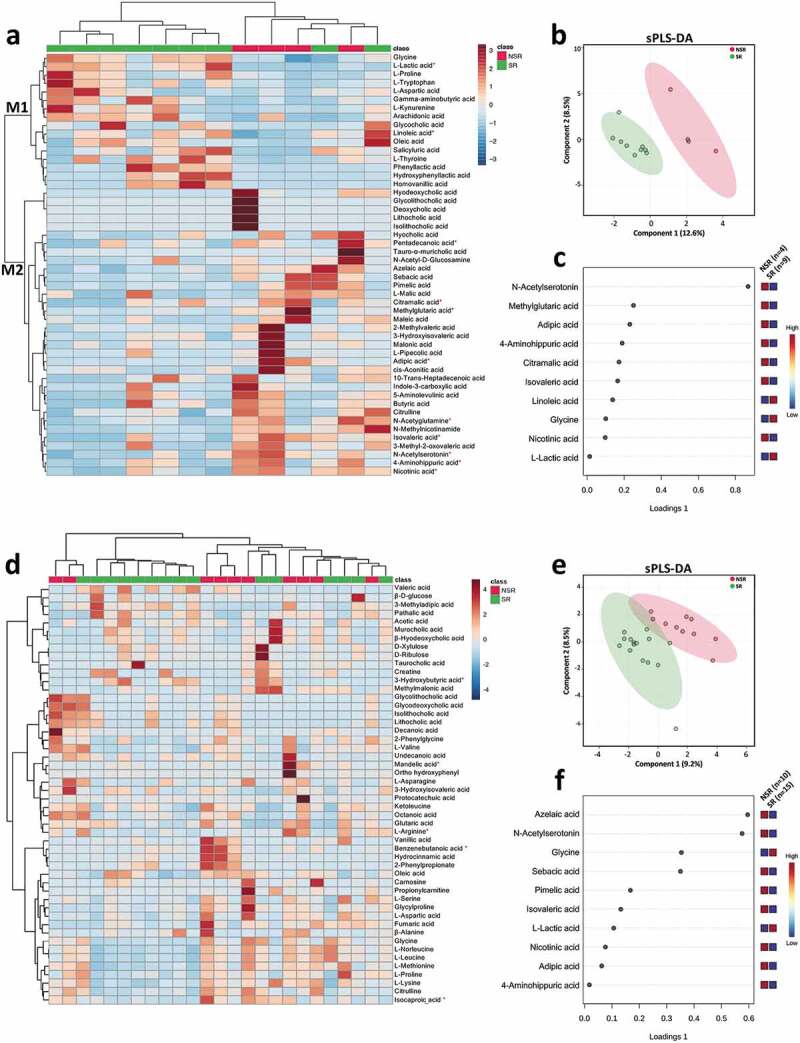
Distinct metabolic profiles are associated with different outcomes before and after IFX treatment. Heat maps showing the top 50 metabolites that different between SR and NSR at baseline (a) and after IFX treatment (d). Significance is determined by using Wilcoxon rank-sum test, with *P < .05. Supervised clustering of fecal metabolites using sPLS-DA in samples of SR and NSR at baseline (b) and after IFX treatment (e). The top metabolites ranked by VIP scores in samples of SR and NSR at baseline (c) and after IFX treatment (f). IFX, infliximab; NSR, non-sustained response; SR, sustained response; sPLS-DA, sparse partial least squares discriminant analysis; VIP, variable importance in projection

Interestingly, we observed distinct fecal metabolome profiles between the pediatric CD patients with different therapeutic outcomes before IFX treatment (Figure 7a, b). There was a pattern in fecal metabolome profiles of the pediatric CD patients with SR in prior to IFX treatment (cluster M1 in Figure 7a), which was featured by relatively higher fecal concentrations of glycine, linoleic acid, and L-lactic acid (Figure 7c). Among the metabolites that enriched in the NSR patients (cluster M2 in Figure 7b), the characteristic metabolites included *N*-acetylserotonin, methylglutaric acid, adipic acid, 4-aminohippuric acid, citramalic acid, isovaleric acid, and nicotinic acid (Figure 7c).

We further identified distinct fecal metabolome profiles between the pediatric CD patients with different therapeutic outcomes after IFX treatment (Figure 7d, e). Higher concentrations of glycine and L-lactic acid were found in the SR patients, whereas the NSR patients showed higher fecal levels of azelaic acid, *N*-acetylserotonin, sebacic acid, pimelic acid, isovaleric acid, nicotinic acid, adipic acid, and 4-aminohippuric acid (Figure 7f).

## Discussion

The components of microbial communities and their metabolites regulate host energy metabolism, immune homeostasis, and mucosal integrity.^30^ The alterations in gut microbiome and metabonomics have been described in many immune-mediated diseases, including IBD.^15,30^ In this study, we characterized the gut microbiota dysbiosis and the altered metabolome in pediatric CD patients, and explored their interactions involved in the therapeutic effects of IFX. The gut microbiota dysbiosis in children with CD was characterized by specific taxonomic alterations in the fecal microbial community of both bacteria and fungi. Agreeing with previous findings,^8–12^ the CD children showed reduced relative abundances of *Bifidobacterium, Blautia, Clostridium IV, Faecalibacterium, Clostridium XIVb, Romboutsia, Roseburia*, and *Turicibacter*, and enhanced relative abundances of *Clostridium XI, Enterococcus, Escherichia Shigella*, and *Peptostreptococcus*. In addition, emerging evidence suggests a role of fungal microbiota in CD pathogenesis in both adult and pediatric populations.^31^ Several studies^7,13,14^ that included small number of patients reported a mycobiota dysbiosis in pediatric CD patients. Our results showed that the mycobiota of pediatric CD patients were characterized by higher relative abundances of *Alternaria* and *Thielavia*, and lower relative abundances of *Botryotinia, Coprinellus, Eupenicillium*, and *Wicherhamomyces* than that of HS.

Metabolites, small molecules that are derived from bacterial metabolism of dietary substrates, modification of host molecules, or directly from bacteria, are key mediators of microbiota–host interactions.^15,32^ Microbial metabolites are detectable in different types of biofluids, such as serum, urine, and blood. Previous metabolomics studies have revealed specific metabolic signatures that discriminate between IBD patients and healthy individuals.^33,34^ However, these studies were heterogeneous for including different subtypes of IBD and using different metabolomics approaches. So far, limited metabolomics studies were performed in pediatric IBD patients.^16–18,35^ Previous fecal metabolomic profiling studies showed that the metabotype associated with pediatric CD was characterized by increased BAs, taurine, and tryptophan.^35^ It has been shown that the serum levels of neopterin, L-arginine, and dimethylglycine were upregulated, while kynurenic acid and trimethylamine-*N*-oxide were downregulated in pediatric CD patients as compared to healthy children.^18^ Ni et al.^36^ showed that fecal amino acids and their derivatives were positively correlated with the intestinal inflammation in children with CD, including proline, phenylalanine, and leucine that confirmed in this study. Overall, our data revealed that the fecal metabolome of the pediatric CD patients was remarkably altered, which were featured by higher levels of several amino acids (L-leucine, L-norleucine) and organic acids (succinic acid, methylmalonic acid), lower levels of SCFAs, and reduced unconjugated/conjugated BAs ratio.

SCFAs, such as acetate, propionate, and butyrate, are an important fuel for intestinal epithelial cells and play a vital role in maintaining the gut barrier function.^37^ Moreover, SCFAs, especially butyrate, are known to exert immunomodulation effects via expanding the pool of intestinal regulatory T cells,^38^ and maintain epithelial homeostasis through the production of interleukin (IL)-18 via inflammasome activation.^39^ The fecal level of SCFAs and gut bacteria ferment fibers that produce SCFAs were typically reduced in IBD patients.^40,41^ As expected, our data demonstrated that all the major SCFAs including acetic acid, butyric acid, propanoic acid, and some SCFA-producing bacteria taxa, such as genera *Faecalibacterium* and *Roseburia*, were reduced in the pediatric CD patients compared to the HS. The deficiency of SCFAs made them to be tested as potential therapeutic agents in treating IBD. However, different approaches, including enemas of butyrate, or mixtures of acetate, propionate, and butyrate resulted in diverse clinical outcomes in IBD patients.^42,43^

There was a dysmetabolism of BA in the pediatric CD featured by a significant reduction in the secondary BA concentrations and unconjugated/conjugated BA ratio, which were likely caused by a loss of bacteria expressing BSH and 7α-dehydroxylase from our 16S rRNA data. This phenomenon agrees with the analysis of metagenomic samples from the Human Microbiome Project and MetaHit, in which reduction in the abundance of a cluster of *bsh* genes was identified in IBD that associated with certain members of Firmicutes phylum.^44^ In fact, BA and the gut microbiota exert bidirectional effects on each other. BA can influence the mucosal barrier integrity, and perform antimicrobial activity by inducing genes that encode anti-bacterial peptides and lectins via farnesoid X receptor (FXR).^45^ Alteration in BA metabolism can cause membrane damage to both microbial cells and the intestinal barrier, and impair the development of colonic RORγ^+^ regulatory T cells thereby promoting intestinal inflammation.^46,47^ Taken together, our data indicate that gut microbiota dysbiosis may contribute to the inflammation and mucosal barrier damage in CD by altering the intestinal metabolome. How this altered metabolic state interacts with the gut microbiota (e.g., maintain microbiota dysbiosis by suppressing certain taxa through anti-bacterial peptides) remains to be illuminated.

It is of clinical value to identify biomarkers for predicting the response of anti-TNF therapy in the clinical management of CD. Recent studies suggested that the changes of gut microbiota were associated with treatment success of anti-TNF therapy in CD patients.^12,22,23,27^ It was shown that elevated proportions of Lachnospiraceae and *Blautia* in feces at week 6 after IFX treatment were associated with the clinical and endoscopic response to IFX in adult CD patients.^48^ Clostridiales was found to be significantly increased in CD patients responding to IFX therapy, which can predict the treatment effectiveness with 86.5% accuracy.^49^ Gut microbiome analysis from mucosal biopsy samples of adult CD patients showed a high abundance of *Hungatella, Ruminococcus gnavus*, and *Parvimonas* at baseline typifies responsive patients to IFX, whereas high abundances *of Blautia, Faecalibacterium, Roseburia*, and *Negativibacillus* genera are associated with disease refractory.^50^ Our data showed that the IFX responders were characterized by higher abundances of *Methylobacterium, Sphingomonas, Staphylococcus*, and *Streptococcus* at baseline, while *Clostridium XI, Clostridium XVIII, Eggerthella, Lachnospiracea incertae sedis, Parabacteroides*, and *Peptococcus* were more abundant in the non-responders. In addition, we further confirmed that the abundances of several SCFA-producing bacteria were increased after IFX treatment in the pediatric CD patients. Above inconsistent results generated from different studies may be due to the limited size, different study population, and large age differences across the study subjects.

In addition to gut bacteria, mycobiota analysis revealed that two fungal genera were potentially associated with the IFX response. A lower relative abundance of *Chaetomium* and a higher abundance of *Malassezia* were identified in the SR patients after IFX treatment. *Malassezia* genus has recently been associated with IBD. For example, *Malassezia restricta* was found to be enriched in the mucosa of CD patients, which may exacerbate colitis via producing inflammatory factors including TNF-α and IL-8.^51^ *Malassezia sympodialis* has been found with a decreased abundance in flare status of CD patients^6^ and increased in those with remission.^52^ Identifying specific species of *Malassezia* may further our understanding of their roles in CD and associations with response to IFX.

Recent studies have suggested that metabolites involving lipid, BAs, and amino acid pathways may contribute to predict response of anti-TNF therapy in adult IBD.^24,26^ Bjerrum et al.^53^ found distinct fecal metabolome profiles between IBD patients with different IFX outcomes, however, no applicable response biomarkers could be identified. Ding et al.^26^ demonstrated that a range of metabolic biomarkers involving amino acid, BA, and lipid pathways may have a potential to predict the response of CD patients to anti-TNF therapy. In particular, serum levels of phosphocholines, ceramides, sphingomyelins, triglycerides, serum and fecal BA, histidine, and urinary cysteine were found to be associated with non-response to anti-TNF therapy in adult CD patients.^26^ However, none of the proposed metabolic biomarkers were identified in our data. The inconsistent findings may be partially explained by a great difference in the age of patients between the two studies. We showed that metabolic phenotypes associated with SR were characterized by higher levels of glycine, linoleic acid, and L-lactic acid in prior to IFX treatment compared to NSR. Neverthless, due to the inconsistent results of limited available studies and small size of our study, the roles of metabolites in predicting anti-TNF therapy in IBD are needed to be further investigated.

There are several limitations that exist in this study. First, although this is the largest longitudinal cohort study of pediatric CD using metabonomic and metataxonomic profiling from China to date, the subgroups within this cohort were small, which may lead to unintentional bias, a discovery-validation cohort study is needed to validate the findings in the future. Second, metabolome and microbiome analysis performed only on fecal samples collected from two time points after IFX treatment that may dilute the time-dependent effect of IFX, and the inadequate sequencing of samples from patients with severe diarrhea may cause a possible bias despite no significant difference was obtained by comparing the serum inflammatory factors in patients with diarrhea to those with normal/lose stools at baseline (data not shown). Third, metatranscriptomic analysis is needed to further investigate the proposed mechanistic relationships between gut microbial community dynamics and metabolic changes.

In summary, the gut microbiota dysbiosis in pediatric CD likely contribute to inflammation and mucosa damage by altering the intestinal metabolism featured by reductions in SCFAs concentrations and an imbalance of unconjugated/conjugated BA ratio. The severity of CD and the outcomes of IFX therapy are correlated with the abundances of certain gut bacteria genera and levels of metabolites. Higher fecal levels of multiple amino acids are a common feature shared by the pediatric CD patients with SR before IFX treatment, which may further be assessed as potential prognostic biomarkers with a large study cohort.

## Methods

### Study design, subjects, and samples collection

This study was approved by the Regional Ethical Review Board in the Shanghai Children’s Hospital and the Regional Ethical Review Board in the Shanghai Tenth People’s Hospital of Tongji University. Written informed consent was obtained from parents or legal guardians of all pediatric participants. Twenty-nine children with CD were prospectively recruited to the study cohort from Department of Gastroenterology, Hepatology and Nutrition, the Shanghai Children’s Hospital, and Department of Gastroenterology, the Shanghai Tenth People’s Hospital of Tongji University, Shanghai, China, between September 2014 and October 2019 (Table 1).

Inclusion criteria were newly diagnosed CD children with age <16 y. The diagnosis of CD was based on the Porto criteria and active disease was measured by the Pediatric Crohn’s Disease Activity Index (PCDAI).^54^ The participating children were monitored prospectively for infections, use of drugs (antibiotics in particular), and other life events. Blood samples were collected to assess erythrocyte sedimentation rate (ESR), white blood cells (WBC), platelets (PLT), C-reactive protein (CRP), albumin (ALB), hemoglobin (HB), and hematocrit (HCT). Exclusion criteria included prebiotics/probiotics supplementation, or the use of antibiotics within the last 3 months prior to inclusion. IFX was administrated via intravenous infusion of 5 mg/kg at weeks 0, 2, and 6, then followed by maintenance intravenous infusions every 8 weeks.^55^ Serum IFX level monitoring was performed during clinical management of the pediatric CD patients with IFX therapy. IFX dosage was increased to the maximum of 10 mg/kg in patients who showed inadequate serum IFX level. All pediatric CD patients included in this study had adequate serum IFX level (3–7 μg/mL) during the study period.

Twenty pediatric healthy subjects (HS) for health check with age and gender matched to CD children, and without previous history of chronic disease were enrolled in the study from the Shanghai Children’s Hospital (Table 1). None of the HS had clinically relevant IBD or allergy symptoms at the time of fecal sample collection, and individuals who took antibiotics within 3 months before fecal sample collection, or any inflammatory conditions were excluded from the study.

Fecal samples for bacteria, fungi, and metabolites analysis were obtained from all participants. Each HS individual provided a single stool sample. A total of 49 fecal samples were collected from the CD patients, including 24 samples at baseline (BSL, prior to IFX treatment) and 25 samples after the third or sixth time of IFX administration (Table S1). All the stool samples were stored at −80°C until DNA extraction and sequencing.

### Genomic DNA extraction

Genomic DNA was extracted using the QIAamp DNA Stool Mini Kit (Qiagen, Germany) combined with the bead-beating method as suggested in the International Human Microbiome Standards (IHMS) protocol Q. In brief, 250 μL of the fecal samples was transferred to a 2 mL tube containing 0.3 g of 0.1 mm zirconia beads (BioSpec, USA), and homogenized for 5 min at 1,500 *g* on a Scientz-48 High-throughput Tissue Grinder (Scientz, China). The eluted DNA was quantified using a dsDNA HS assay on a Qubit 3.0 (Thermo Fisher Scientific, USA). The DNA concentration of each sample was adjusted to 50 ng/μl for subsequent 16S rRNA/ITS2 gene sequencing.

### PCR amplification

Isolated genomic DNA was amplified for the 16S rRNA V3-V4 hypervariable regions (approximately 465 bases) using the universal primer set 341 F 5ʹ-CCTACGGGAGGCAGCAG-3ʹ/806 R 5ʹ-GGACTACHVGGGTWTCTAAT-3ʹ. ITS2 primers (Forward 5ʹ-GTGARTCATCGAATCTTT-3ʹ/Reverse 5ʹ-GATATGCTTAAGTTCAGCGGGT-3ʹ) were used to amplify the ITS2 gene fragment (approximately 350 bases). For each sample, 25 μL PCR reactions were performed using 10 ng template genomic DNA, 0.5 μM of forward/reverse primers, and 1X Phusion® High Fidelity Buffer. Genomic DNA was initial denatured at 98°C for 1 min, followed by 30 cycles of denaturation at 98°C for 10 s, annealing at 50°C for 30 s, elongation at 72°C for 30 s, and a final extension step at 72°C for 5 min. The PCR products were purified using QIAquick Gel Extraction Kit (Qiagen, Hilden, Germany). Bar-coded libraries were generated using TruSeq® DNA PCR-Free Sample Preparation Kit (Illumina, USA) as per the manufacturer’s recommended protocol. Library quality was assessed on a Qubit 2.0 Fluorometer (Thermo Scientific, USA), and the library concentration was estimated on an Agilent 2100 Bioanalyzer (Agilent Technologies, USA). Each library was diluted to a final concentration of 12.5 nM and pooled in an equimolar ratio prior to clustering. Sequencing was performed using an Illumina NovaSeq platform (Illumina, USA), and 2 × 250 bp paired-end reads were generated.

### Gene sequencing analysis

Quality control check on raw sequence data was performed using FastQC (Babraham Bioinformatics, UK). Post-processing of the sequencing reads was performed using USEARCH (version 10.0.240).^56^ Raw forward and reverse reads were joined, and quality trimmed by using -*fastq_filter* command with -*fastq_maxee* 1.0. The unique sequences in FASTA format were generated by using *fastx_uniques* command. NOISE3 algorithm was used to remove chimeras in the unique sequences, and produce representative zero-radius operational taxonomic units (OTU) for subsequent analyses.^57^ Ribosomal Database Project (RDP) reference database was employed for taxonomic assignment.

Calypso (version 8.84) was used to analyze the bacterial and fungal community composition data.^58^ The OTU counts were normalized by total sum normalization (TSS). Taxa have less than 0.02% relative abundances were excluded from the following analysis. Cumulative-sum scaling (CSS) was applied for correcting biases introduced by TSS, followed by log2 transformation to account for the non-normal distribution of the taxonomic counts data. The microbiome alpha diversity was measured using the Shannon’s, Chao1, Simpson’s, and Inverse Simpson’s indexes. The beta diversity among samples were calculated through Principal coordinate analysis (PCoA) to Bray–Curtis distance based on the OTU abundance. Permutational multivariate analysis of variance (PERMANOVA) was carried out to test whether the gut microbiome structure was significantly different between two groups. Statistical differences in alpha diversity and the abundances of taxa were assessed by Wilcoxon rank-sum test for comparison between two groups with *P* values adjusted for False Discovery Rate (FDR).

### Quantification of fecal metabolites

Targeted metabolomics analysis was performed to measure the absolute concentrations of fecal metabolites as previously described.^59,60^ For each sample, 10 mg of lyophilized homogenized feces was dissolved in 40 µL of water using a Bullet Blender Tissue Homogenizer (Next Advance, Inc., Averill Park, NY). A 200 µL aliquot of methanol containing 50 internal standards was homogenized. After centrifugation, 40 µL supernatant from each sample was transferred to a 96-well plate, and freshly prepared derivative reagents were added to each well. The plate was sealed and the derivatization was carried out at 30°C for 60 min. After derivatization, 300 μL of ice-cold 50% methanol solution was added to dilute the sample. Then, the plate was stored at −20°C for 20 min and followed by 4,000 *g* centrifugation at 4°C for 30 min. A 150 μL of supernatant was transferred to a new 96-well plate for injection. A 5 μL aliquot of sample was injected for liquid chromatography–tandem mass spectrometry analysis measurement, and the flow rate was 0.4 mL/min.

The fecal metabolites were quantified with an ultraperformance liquid chromatography (UPLC) coupled to tandem mass spectrometry (UPLC-MS/MS) system (ACQUITY UPLC-Xevo TQ-S, Waters Corp., Milford, MA, USA). The MassLynx software (version 4.1, Waters, Milford, MA) was used for instrument control, data acquisition and processing. Chromatographic separation was achieved by an ACQUITY UPLC BEH C_18_ column (2.1 mm × 100 mm, 1.7 µm). UPLC-MS raw data obtained with both positive and negative ionization mode were analyzed using TargetLynx™ application manager (Waters Corp., Milford, MA, USA) to obtain calibration equations and the quantitative concentration of each metabolite.

MetaboAnalyst 4.0.^61^ was used for statistics and feature selection of the metabolomic data. Sparse partial least squares discriminant analysis (sPLS-DA) and random forest model was employed for classification and feature selection. Univariate statistical analysis was performed using Wilcoxon rank-sum test with *P* values adjusted with Bonferroni correction procedure.

### Metabolic function network analysis

Metabolic function network analysis was performed in the M2IA server.^62^ Differential metabolites and bacteria between CD and HS at baseline were selected at first according to univariate analysis (Mann–Whitney *U* test, *P* value<.01). The Spearman correlation coefficients were then calculated between the differential metabolites and bacteria using pairwise correlation analysis method. Coefficient values *R* ≤ −0.5 and ≥0.5 with *P* value < .01 were considered statistically significant and were plotted in the network.

## Supplementary Material

Supplemental Material

## Data Availability

Raw metabolomic data for this study are available at the MetaboLights metabolomics repository under study accession number MTBLS1753. Raw sequencing data are available at the European Nucleotide Archive server under study accession number PRJEB38210.

## Abbreviations

AUC, area under the curve

BA, bile acids

BSH, bile salt hydrolases

CA, cholic acid

CD, Crohn’s disease

CDCA, chenodeoxycholic acid

CRP, C-reactive protein

ESR, erythrocyte sedimentation rate

HB, hemoglobin

HS, healthy subjects

IBD, inflammatory bowel disease

IFX, infliximab

IQR, interquartile range

NSR, non-sustained response

OTU, operational taxonomic units

PCDAI, Pediatric Crohn’s Disease Activity Index

PCoA, principal coordinate analysis

PLS-DA, partial least squares discriminant analysis

PLT, platelets

RDP, ribosomal database project

ROC, receiver operating characteristic

SCFAs, short-chain fatty acids

SR, sustained response

TNF, tumor necrosis factor

UC, ulcerative colitis

UPLC, ultraperformance liquid chromatography

VIP, variable importance in projection

WBC, white blood cells
