# Supplementary material for: Microbial and metabolic features associated with outcome of infliximab therapy in pediatric Crohn’s disease
Source: Gut Microbes. 2021 Jan 11;13(1):1865708. doi: 10.1080/19490976.2020.1865708 (PMC7808429; doi:10.1080/19490976.2020.1865708)
Supplement: Supplemental Material [file KGMI_A_1865708_SM6867.zip › supplement/Table for review only.docx]

|  | PCDAI | WBC (×10^9^/L) | PLT (×10^9^/L) | CRP (mg/L) | ESR (mm/h) |
| --- | --- | --- | --- | --- | --- |
| Diarrhea, mean ± SD, (N=7) | 31.86 ± 21.71 | 11.88 ± 5.22 | 401.86 ± 157.61 | 37.1 ± 40.61 | 45.43 ± 40.46 |
| Normal/lose stools, mean ± SD, (N=17) | 27.9 ± 13.86 | 9.29 ± 3.11 | 389.27 ± 105.38 | 34.9 ± 30.45 | 71.07 ± 41.06 |
| Mann Whitney U test P-value | 0.9718 | 0.4475 | 0.6796 | 0.8599 | 0.2177 |

**Table.** Comparison of the serum inflammatory markers in pediatric CD patients with diarrhea to those with normal/lose stools at baseline (Baseline samples were not available from 5 CD patients).

CD, Crohn’s disease; WBC, white blood cells; ESR, erythrocyte sedimentation rate; PLT, platelets; CRP, C-reactive protein; PCDAI, Pediatric Crohn's Disease Activity Index.
