# Supplementary figures and images for: Microbial and metabolic features associated with outcome of infliximab therapy in pediatric Crohn’s disease
Source: Gut Microbes. 2021 Jan 11;13(1):1865708. doi: 10.1080/19490976.2020.1865708 (PMC7808429; doi:10.1080/19490976.2020.1865708)

**a**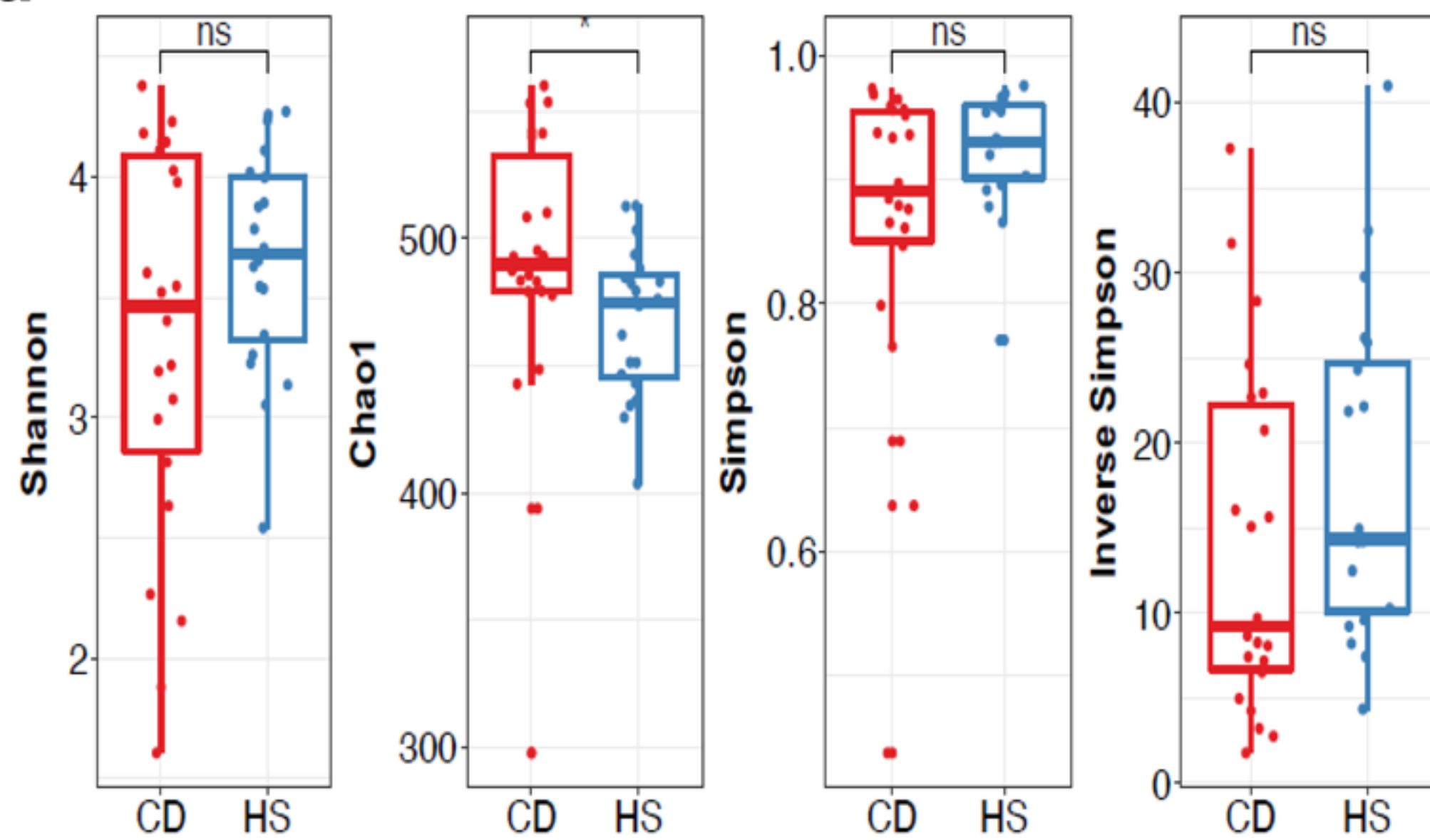**b**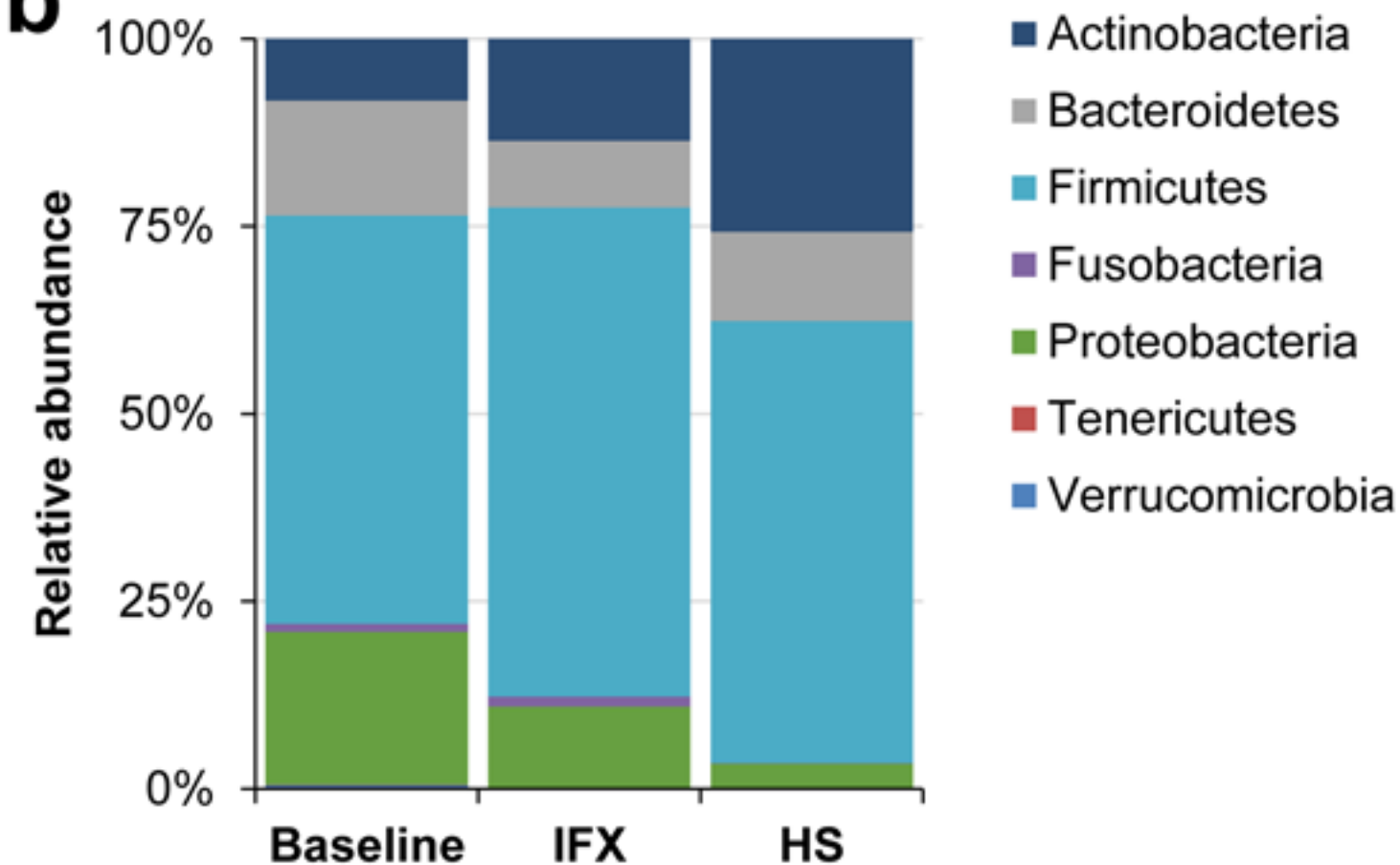**d**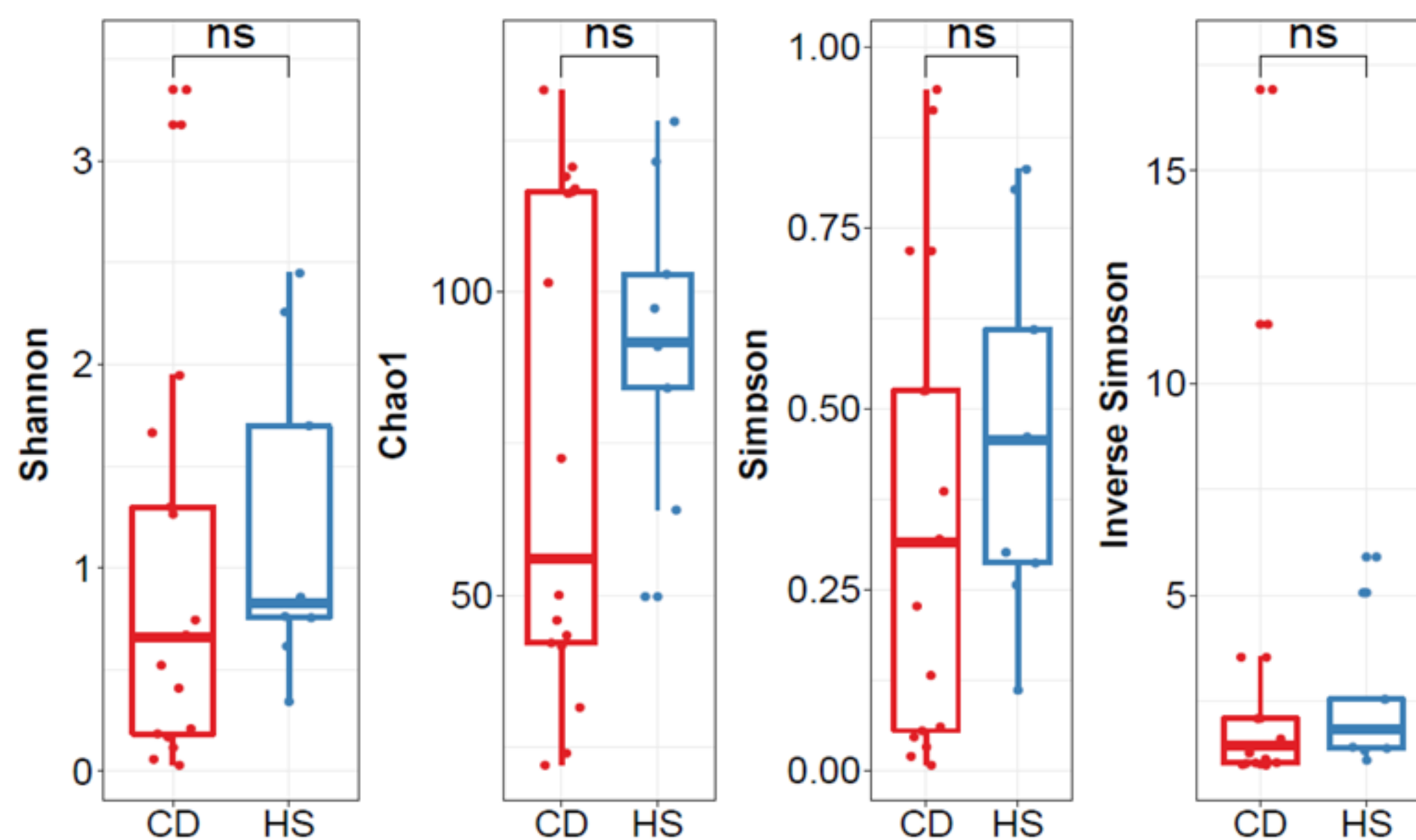**c**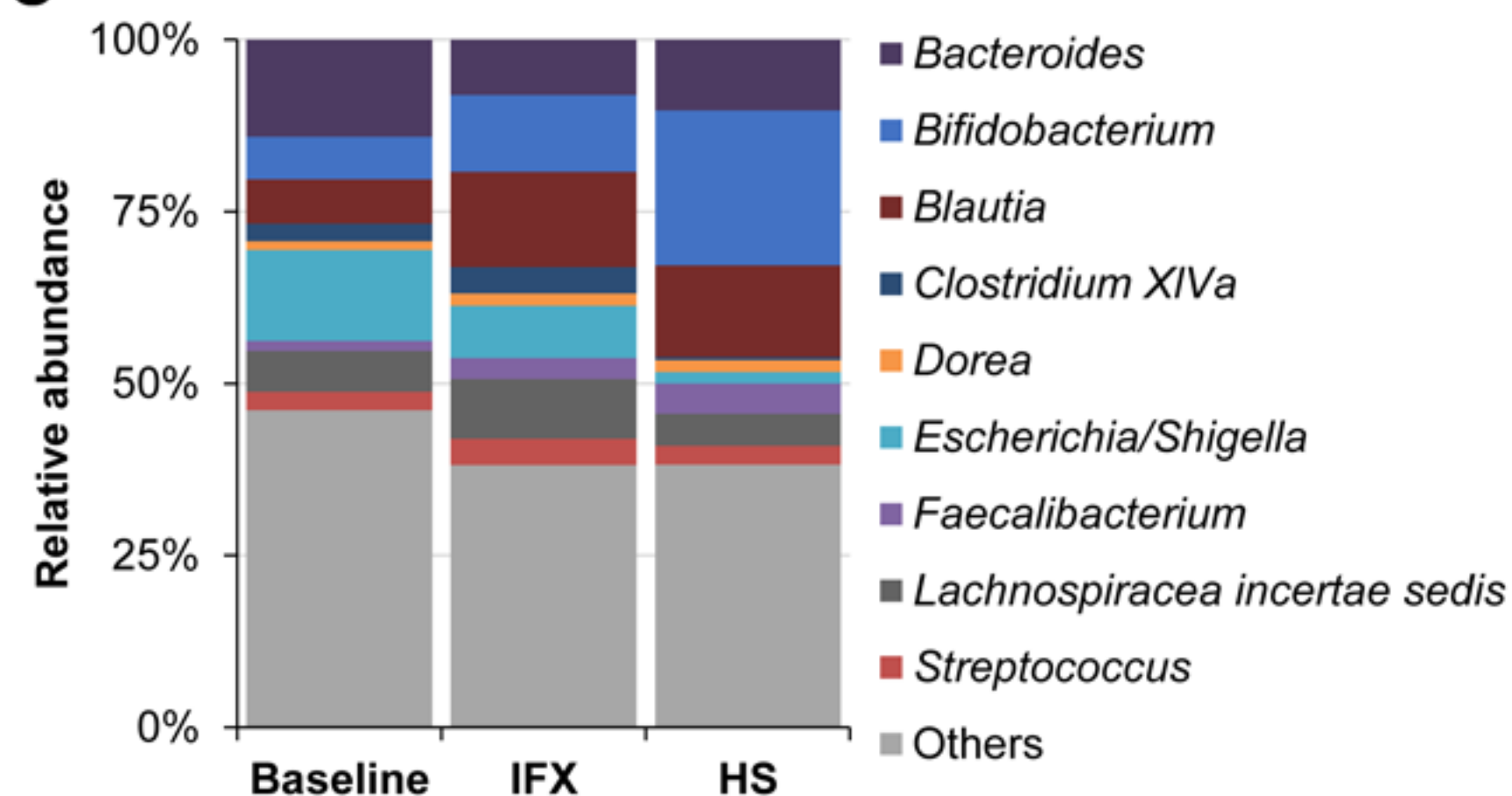**e**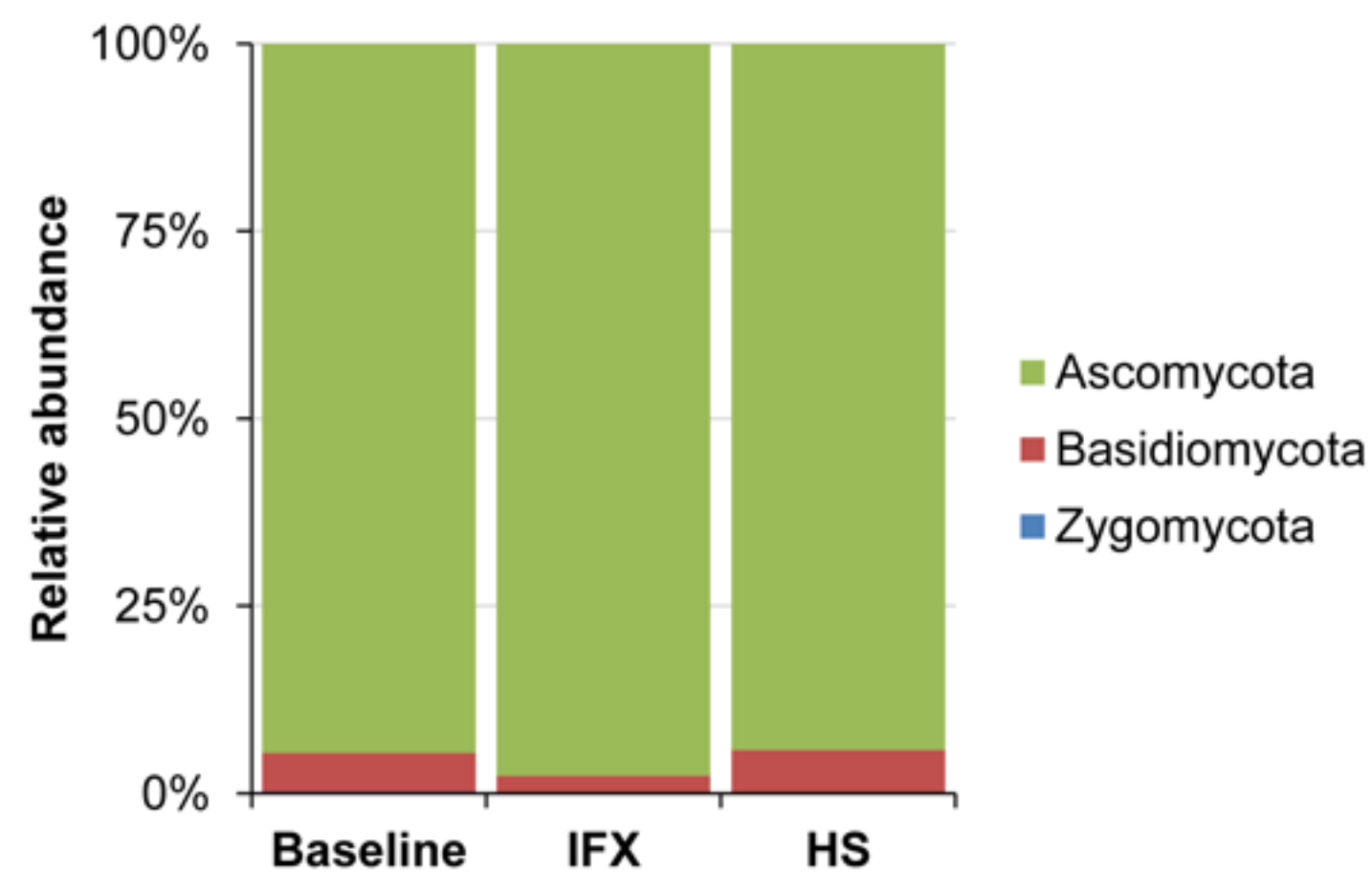**f**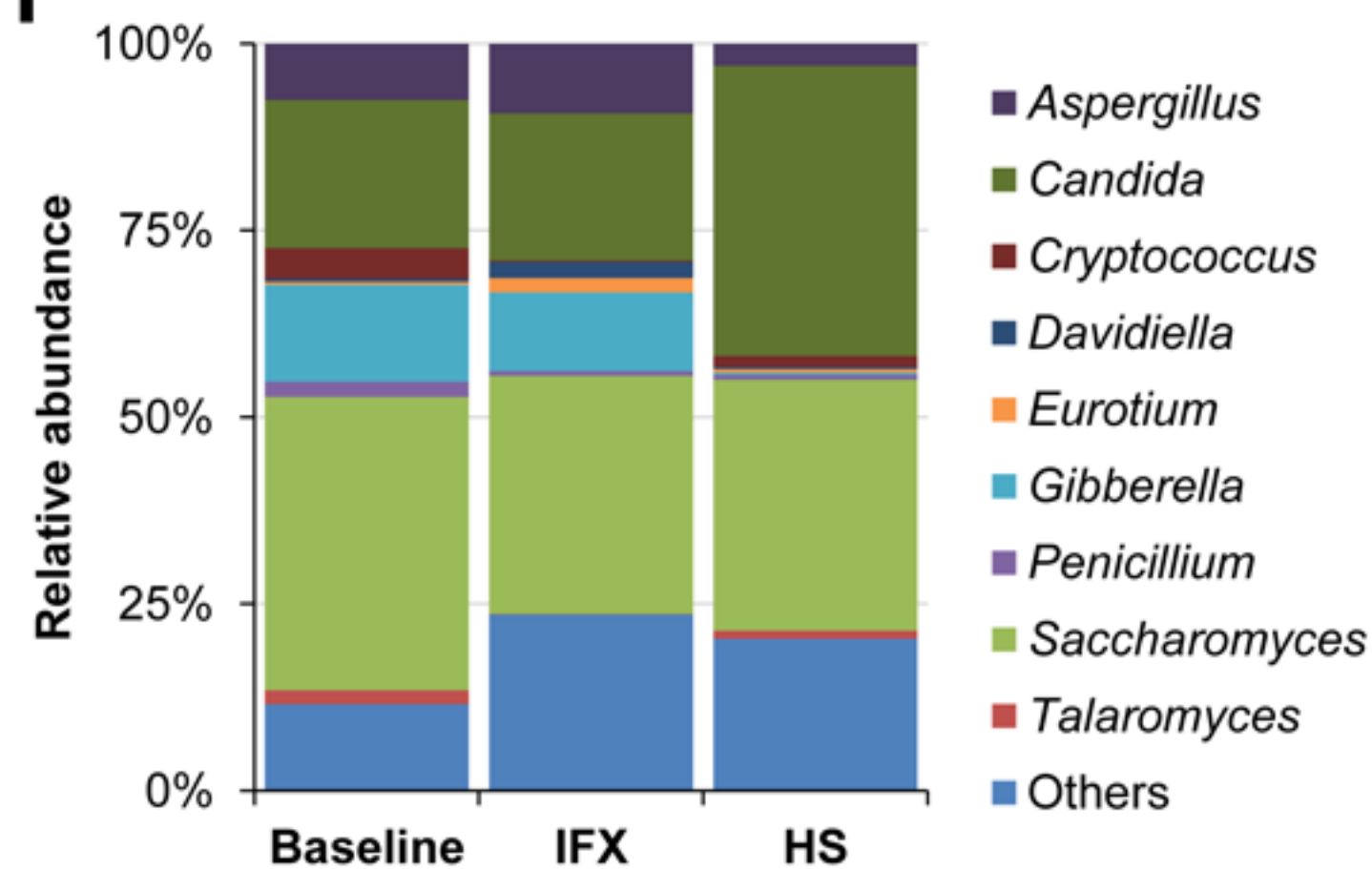

Supplement: Supplemental Material [file KGMI_A_1865708_SM6867.zip › supplement/Figure S1.pdf]

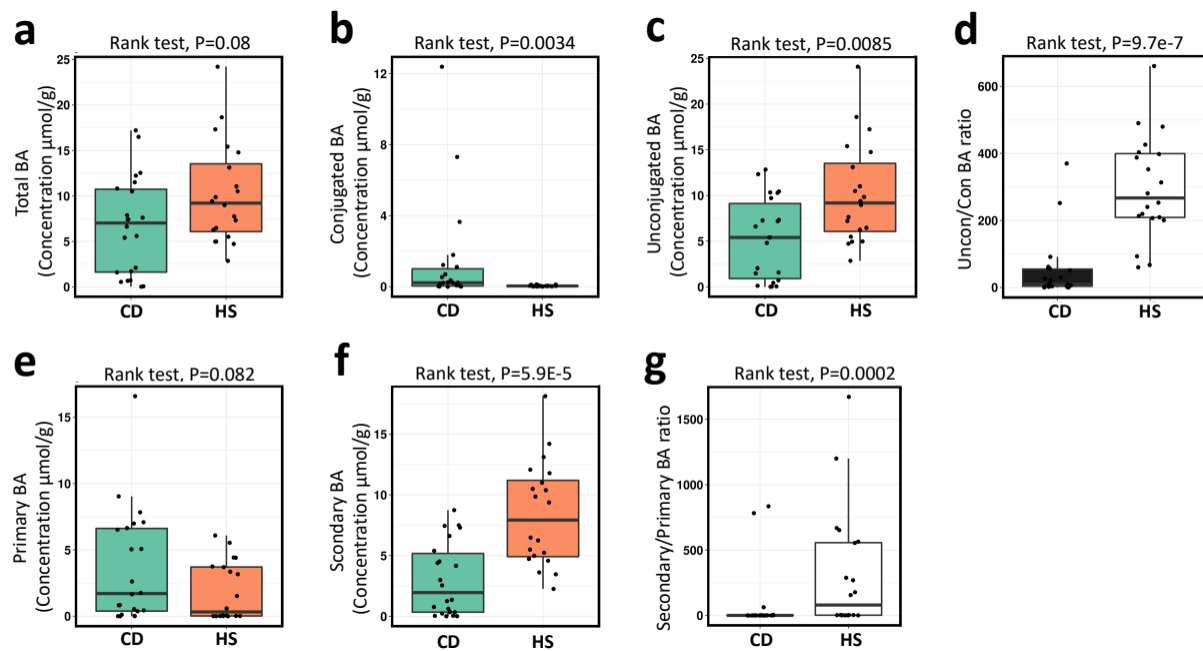

Supplement: Supplemental Material [file KGMI_A_1865708_SM6867.zip › supplement/Figure S2.pdf]

**a**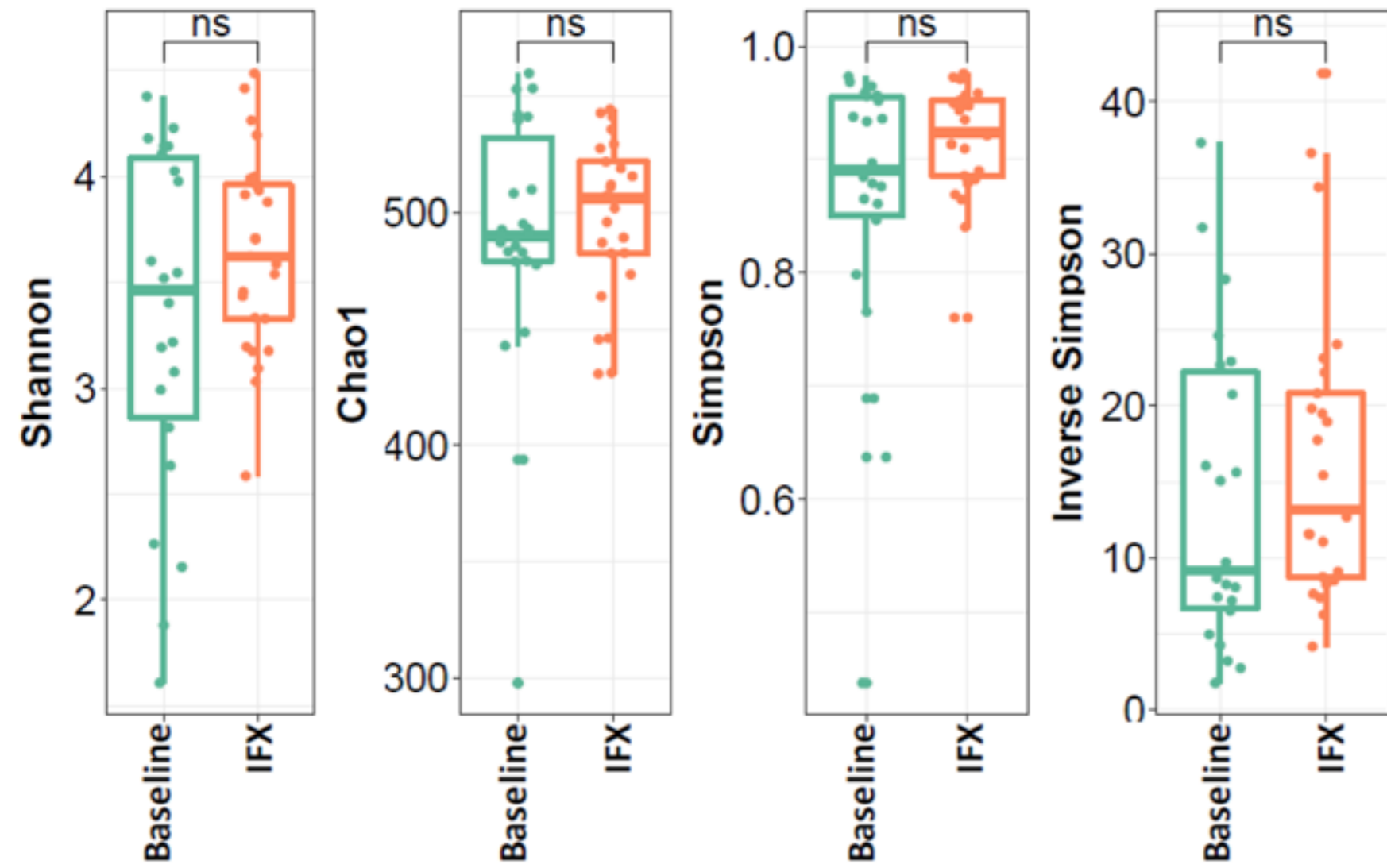**b**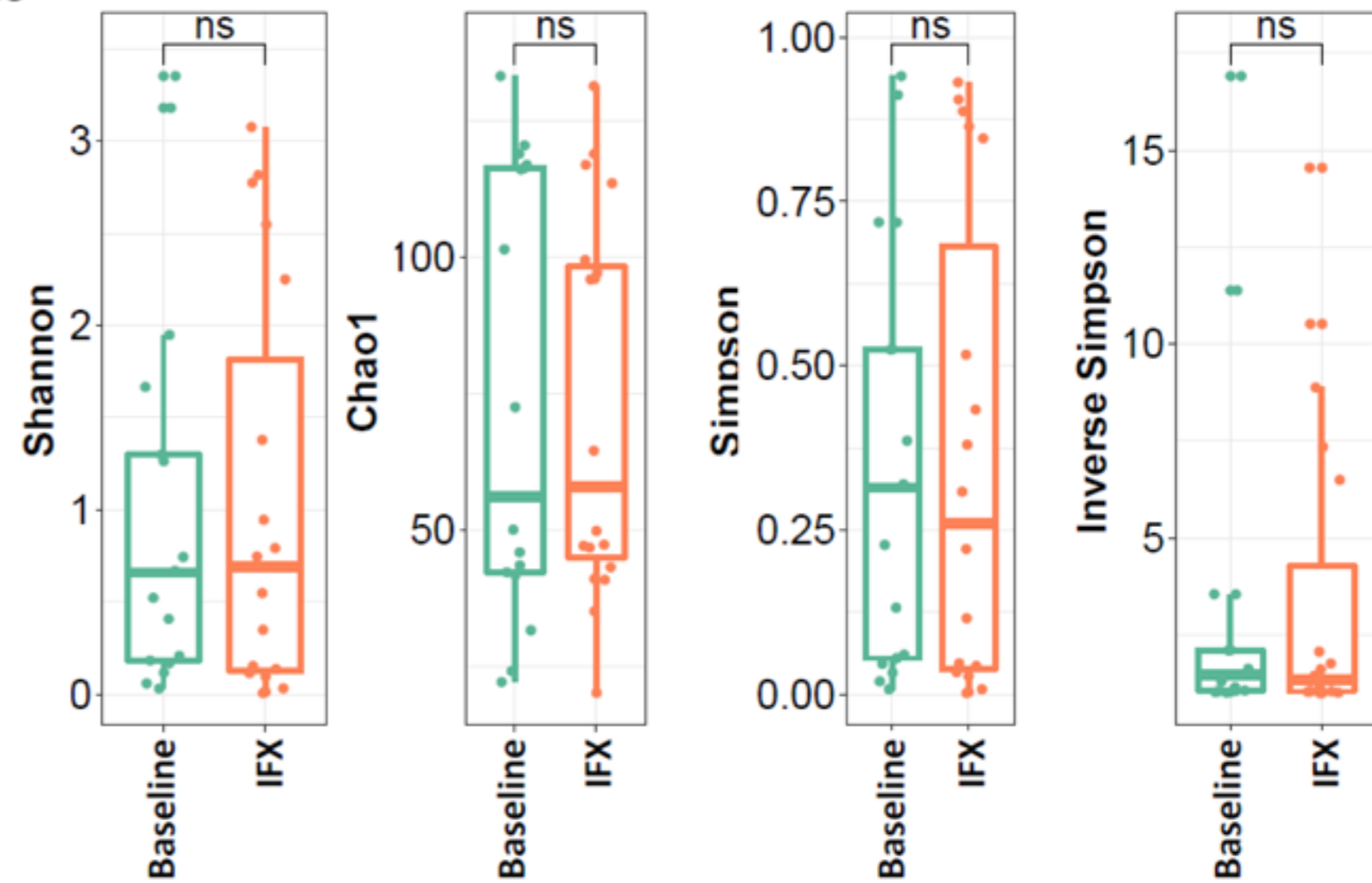

Supplement: Supplemental Material [file KGMI_A_1865708_SM6867.zip › supplement/Figure S3.pdf]

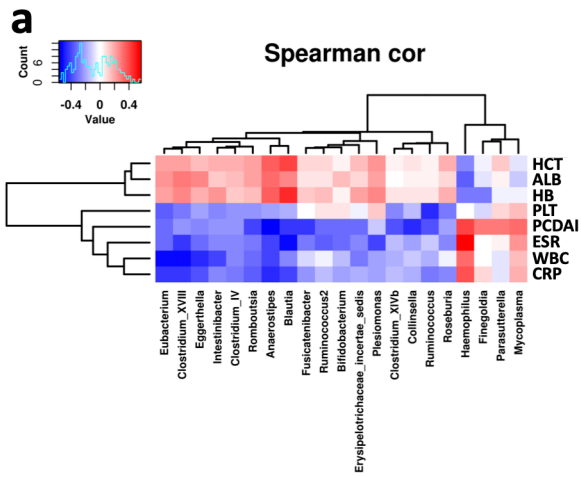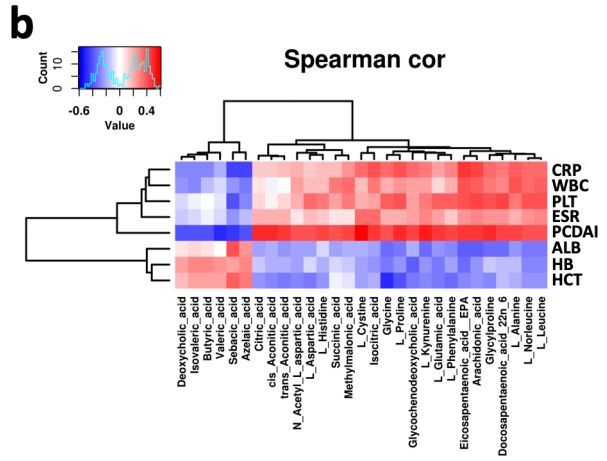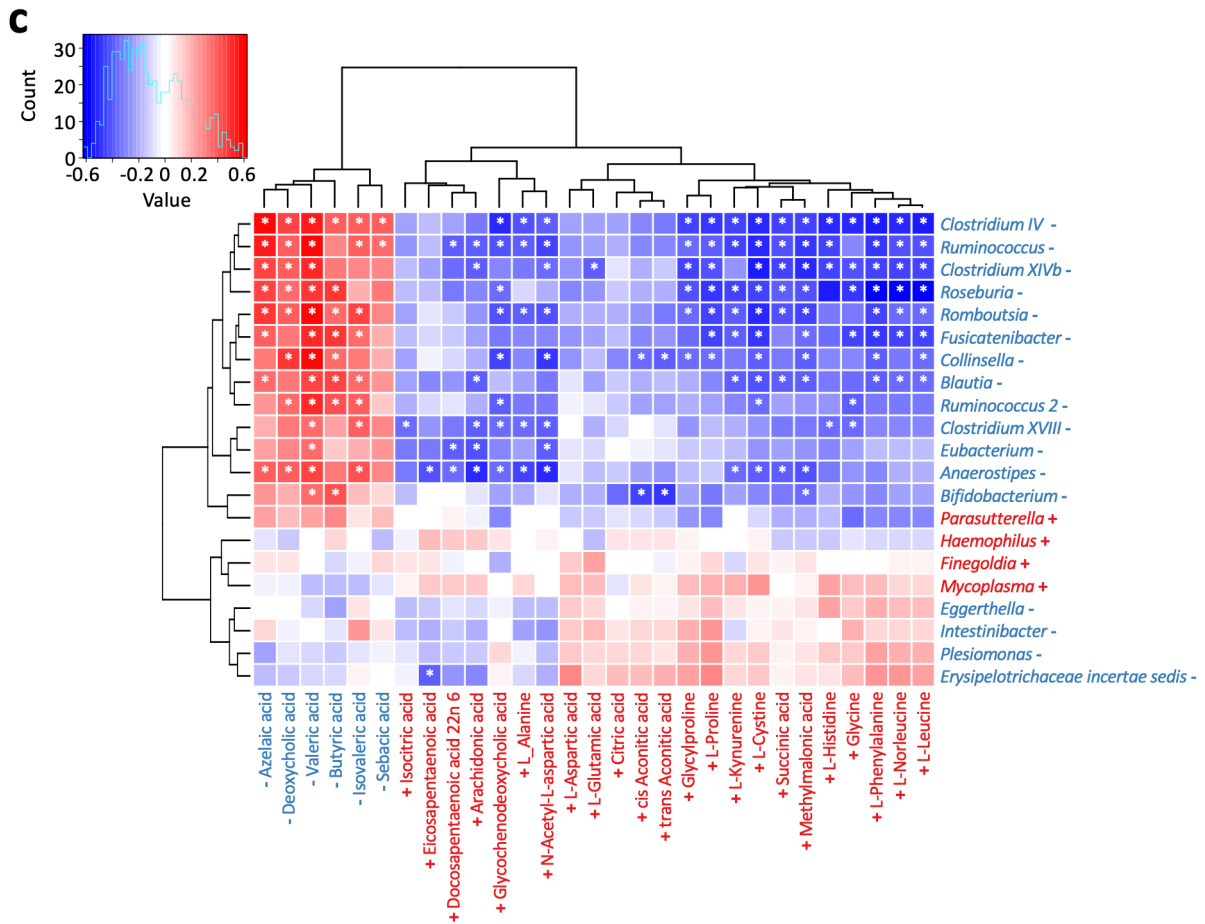

Supplement: Supplemental Material [file KGMI_A_1865708_SM6867.zip › supplement/Figure S4.pdf]

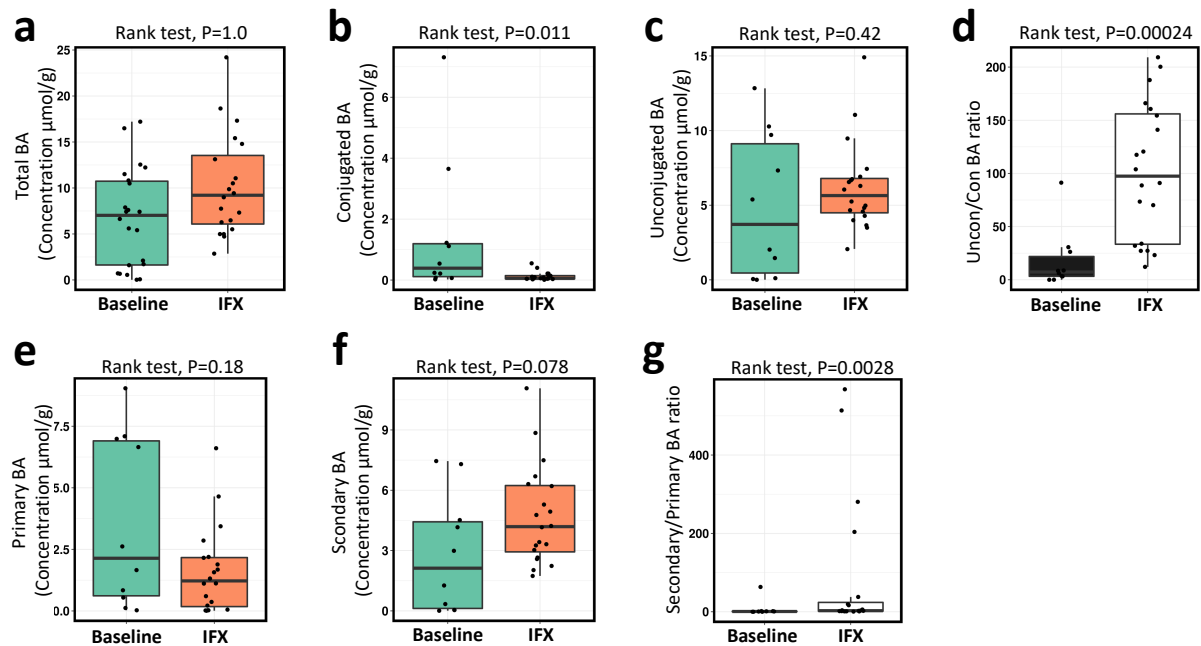

Supplement: Supplemental Material [file KGMI_A_1865708_SM6867.zip › supplement/Figure S5.pdf]

**a**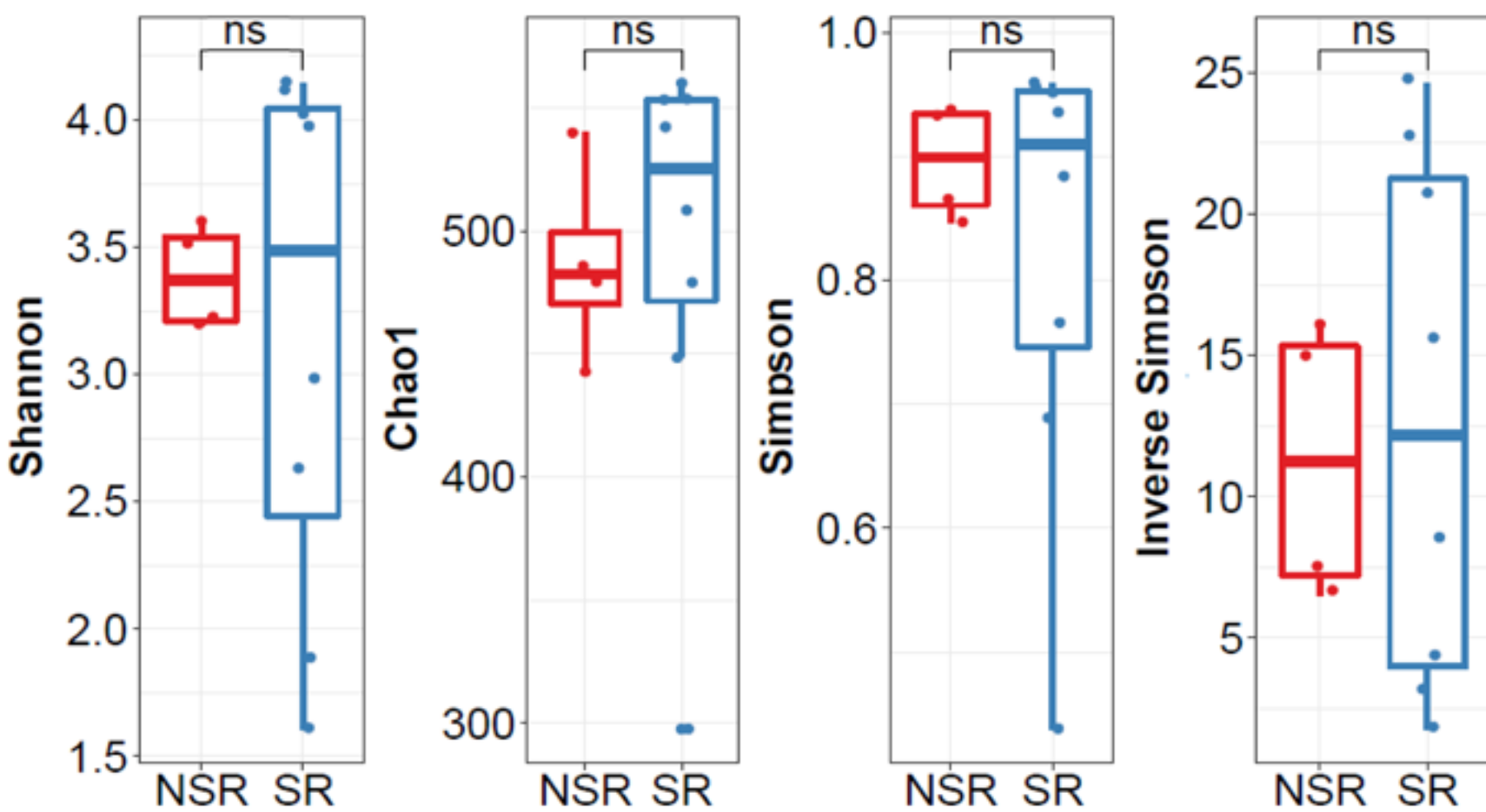**b**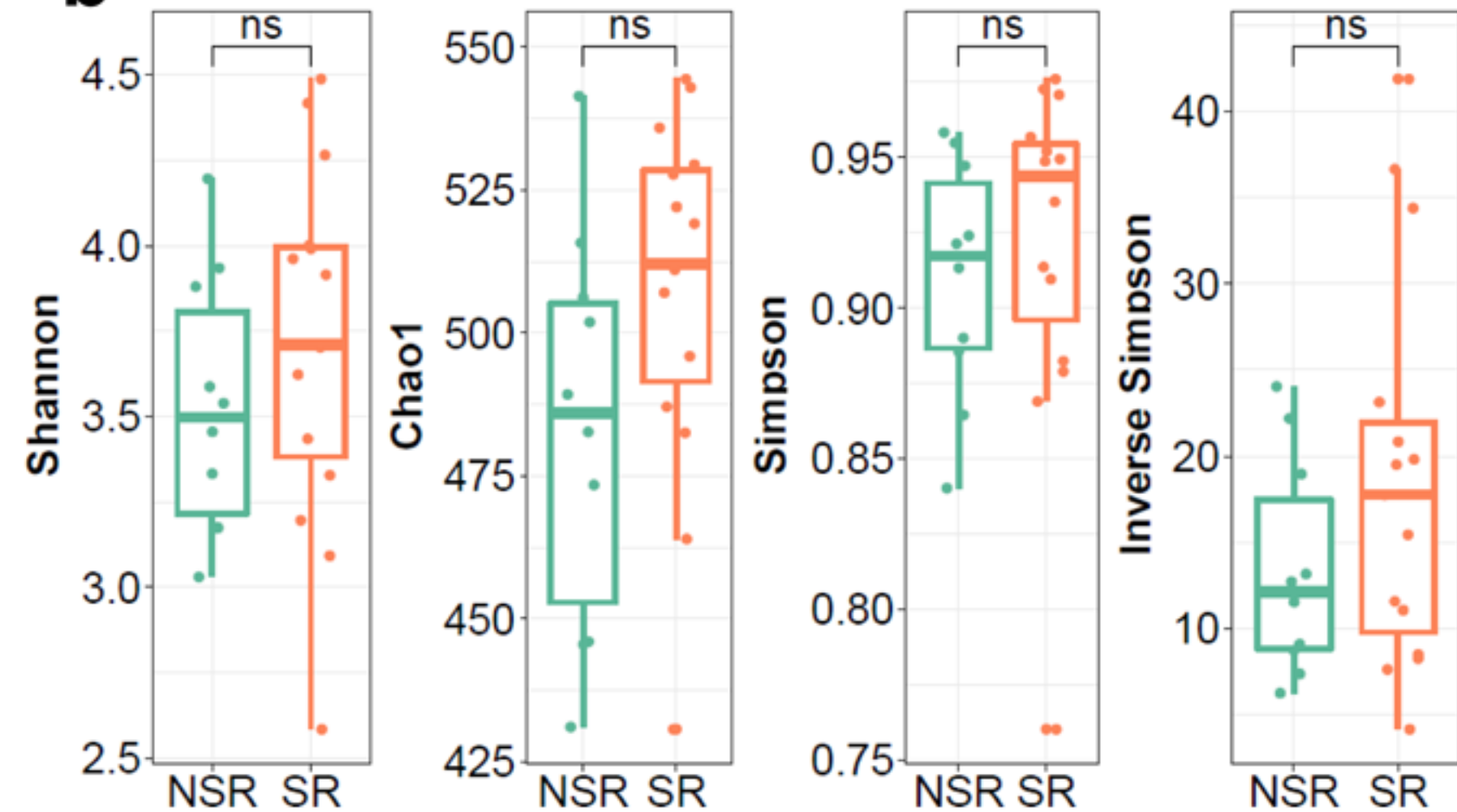**c**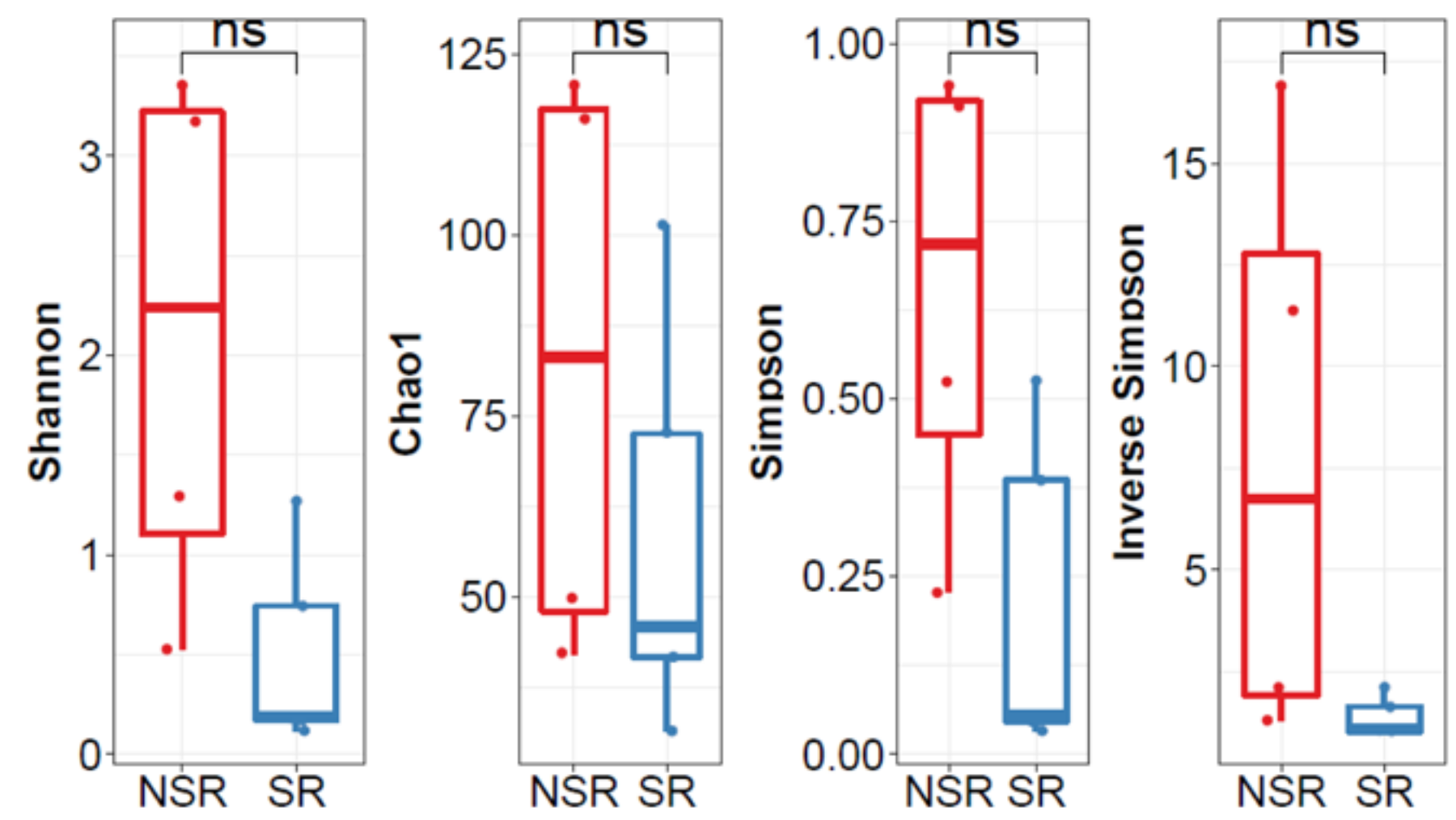**d**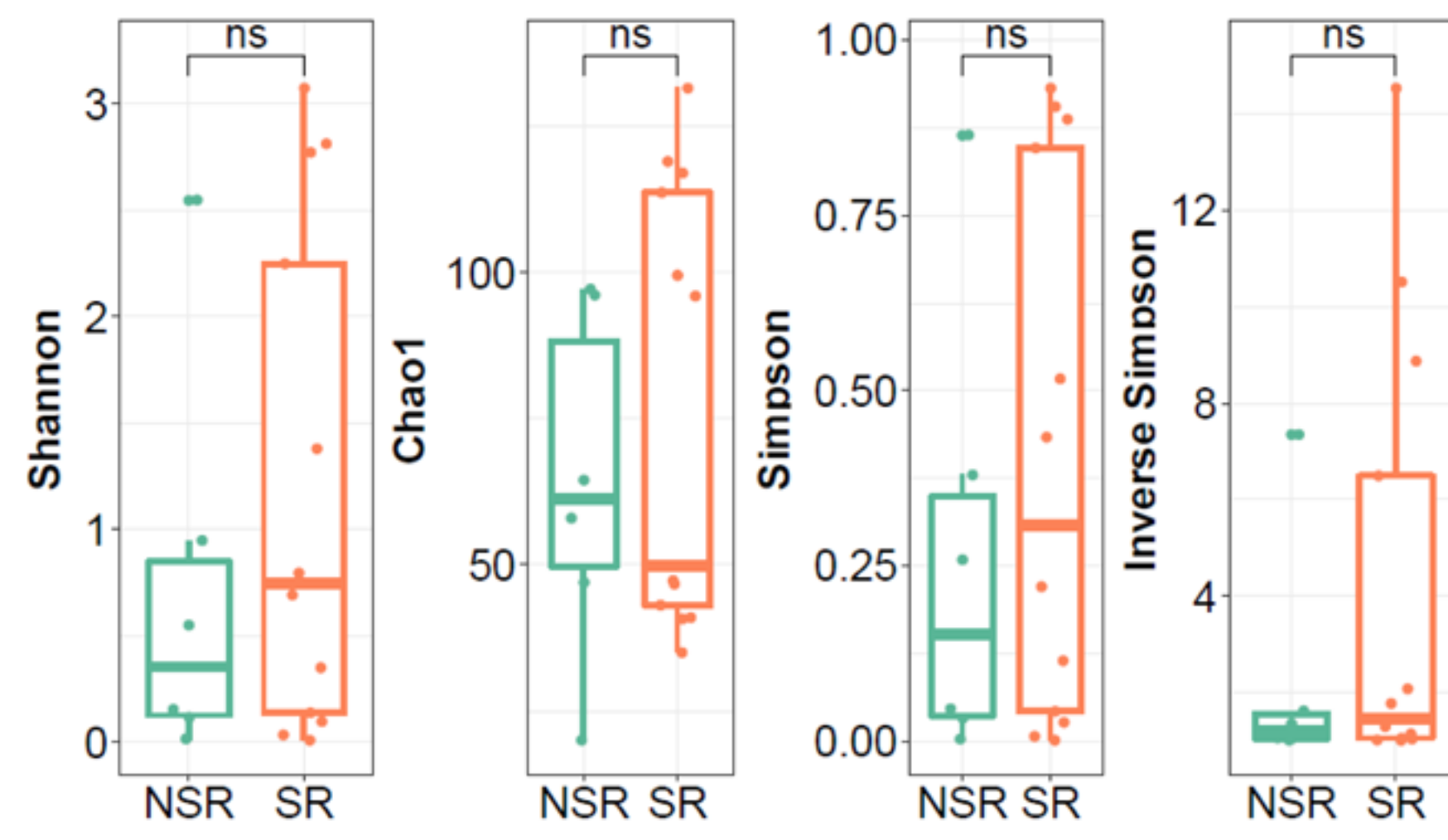

Supplement: Supplemental Material [file KGMI_A_1865708_SM6867.zip › supplement/Figure S6.pdf]
