# Supplementary material for: Microbial and metabolic features associated with outcome of infliximab therapy in pediatric Crohn’s disease
Source: Gut Microbes. 2021 Jan 11;13(1):1865708. doi: 10.1080/19490976.2020.1865708 (PMC7808429; doi:10.1080/19490976.2020.1865708)
Supplement: Supplemental Material [file KGMI_A_1865708_SM6867.zip › supplement/Supplementary Tables 20201201.doc]

**Supplementary Tables**

**Table S1.** Demographic and clinical characteristics of the pediatric CD patients before and during IFX therapy.

| Patient ID | Gender | Age  (Year) | Disease location# | IFX SR/NSR | WBC (×10^9^/L) | | |  | ESR (mm/h) | | |  | PLT (×10^9^/L) | | |  | CRP (mg/L) | | |  | PCDAI | | |
| --- | --- | --- | --- | --- | --- | --- | --- | --- | --- | --- | --- | --- | --- | --- | --- | --- | --- | --- | --- | --- | --- | --- | --- |
|  |  |  |  |  | BSL | 3rd^&^ | 6th |  | BSL | 3rd | 6th |  | BSL | 3rd | 6th |  | BSL | 3rd | 6th |  | BSL | 3rd | 6th |
| CD01 | Female | 9 | L3 | SR | 18.11 | 20.65* | 5.38 |  | 50 | 22 | 2 |  | 515 | 382 | 229 |  | 76 | <5 | <5 |  | 12 | 10 | 0 |
| CD02 | Male | 11 | L3 | SR | 11.47* | 5.80* | 10.7* |  | 104 | 3 | 31 |  | 433 | 256 | 319 |  | 32 | <5 | 8 |  | 37.5 | 0 | 2 |
| CD03 | Male | 6 | L3 | SR | 17.00* | 6.28* | 4.26* |  | 65 | 10 | 14 |  | 531 | 307 | 257 |  | 36 | <5 | <5 |  | 37.5 | 5 | 0 |
| CD04 | Male | 13 | L3 | SR | 7.90* | 7.22 | 6.55* |  | 88 | 8 | 28 |  | 443 | 325 | 321 |  | 13 | <5 | <5 |  | 20 | 5 | 2 |
| CD05 | Female | 14 | L3 | SR | 8.51* | 4.26 | 5.84 |  | 106 | 18 | 10 |  | 540 | 224 | 295 |  | 52 | <5 | <5 |  | 42.5 | 0 | 0 |
| CD06 | Female | 11 | L3 | NSR | 8.27* | 4.94 | 7.50* |  | 64 | 15 | 50 |  | 326 | 239 | 333 |  | 29 | <5 | 13 |  | 20 | 5 | 12 |
| CD07 | Female | 12 | L3 | NSR | 5.55 | 4.99* | 6.16* |  | 106 | 112 | 120 |  | 494 | 400 | 237 |  | 57 | 35 | 35 |  | 40 | 12.5 | 15 |
| CD08 | Male | 11 | L3 | SR | 17.75* | 4.72* | 4.87* |  | 120 | 26 | 26 |  | 573 | 220 | 209 |  | 112 | <5 | <5 |  | 32.5 | 2 | 2 |
| CD10 | Female | 16 | L3 | NSR | 17.80 | 9.30 | 9.50* |  | 54 | 120 | 120 |  | 557 | 446 | 681 |  | 79 | 41 | 71 |  | 35 | 15 | 20 |
| CD11 | Female | 14 | L3 | NSR | 8.03 | 7.60 | 8.50* |  | 120 | 120 | 76 |  | 477 | 402 | 417 |  | 26 | 18 | <5 |  | 57.5 | 22.5 | 25 |
| CD12 | Male | 10 | L4 | NSR | 11.40* | 4.83* | 7.40* |  | 14 | 27 | 10 |  | 437 | 541 | 383 |  | 6 | 11 | 17 |  | 15 | 9.5 | 15 |
| CD13 | Male | 11 | L3 | SR | 14.88* | 6.84* | 6.08* |  | 120 | 9 | 10 |  | 469 | 342 | 227 |  | 72 | <5 | <5 |  | 47.5 | 5 | 5 |
| CD14 | Male | 14 | L2 | SR | 11.96 | 5.38* | 6.70 |  | 97 | 15 | 4 |  | 653 | 288 | 265 |  | 46 | <5 | <5 |  | 42.5 | 0 | 0 |
| CD15 | Male | 15 | L3 | SR | 6.40* | 5.14* | 4.10* |  | 44 | - | 28 |  | 455 | 316 | 244 |  | 58.7 | <5 | <5 |  | 35 | 22.5 | 2 |
| CD16 | Male | 12 | L3 | NSR | 7.17* | 4.18* | 5.27* |  | 28 | - | - |  | 242 | 281 | 231 |  | 6.4 | <5 | <5 |  | 32.5 | 25 | 25 |
| CD17 | Male | 14 | L3 | SR | 9.74* | 5.73* | 8.55* |  | 32 | - | - |  | 289 | 231 | 282 |  | 45.6 | <5 | <5 |  | 25 | 12.5 | 5 |
| CD18 | Male | 12 | L2 | NSR | 4.37* | 4.13* | - |  | 8 | - | - |  | 459 | 234 | - |  | <5 | <5 | - |  | 32.5 | 30 | - |
| CD19 | Male | 15 | L4 | SR | 3.41* | 6.03 | 7.21 |  | 20 | 15 | 9 |  | 306 | 224 | 228 |  | 15.8 | <5 | <5 |  | 27.5 | 12.5 | 5 |

^#^ParisL; *Fecal sample collected; ^&^After n times of IFX infusion. CD, Crohn’s disease; IFX, infliximab; SR, sustained response; NSR, non-sustained response, WBC, white blood cells; ESR, erythrocyte sedimentation rate; PLT, platelets; CRP, C-reactive protein; PCDAI, Pediatric Crohn's Disease Activity Index

**Table S2. Speaman correlation** **coeeficients between fecal bacteria and metabolites identify from healthy subjects and CD patients at baseline.**

|  | **Phylum** | **Genus** | **Metabolite class** | **Metabolite** | **R (Spearman)** | **P** |
| --- | --- | --- | --- | --- | --- | --- |
| **Positive correlations** | Actinobacteria | *Adlercreutzia* | Benzenoids | Phthalic acid | 0.633 | 6.70E-06 |
|  | Actinobacteria | *Adlercreutzia* | Bile acids | isolithocholic acid | 0.682 | 6.70E-07 |
|  | Actinobacteria | *Collinsella* | Bile acids | Lithocholic acid | 0.668 | 1.30E-06 |
|  | Actinobacteria | *Collinsella* | Bile acids | Hyodeoxycholic acid | 0.667 | 1.40E-06 |
|  | Actinobacteria | *Adlercreutzia* | Bile acids | Lithocholic acid | 0.641 | 4.80E-06 |
|  | Actinobacteria | *Collinsella* | Bile acids | isolithocholic acid | 0.633 | 6.80E-06 |
|  | Actinobacteria | *Adlercreutzia* | Bile acids | Hyodeoxycholic acid | 0.632 | 7.20E-06 |
|  | Actinobacteria | *Adlercreutzia* | Fatty acids | 3-Methyladipic acid | 0.659 | 2.10E-06 |
|  | Actinobacteria | *Collinsella* | Fatty acids | Valeric acid | 0.653 | 2.80E-06 |
|  | Actinobacteria | *Adlercreutzia* | Fatty acids | Azelaic acid | 0.641 | 4.70E-06 |
|  | Bacteroidetes | *Alistipes* | Bile acids | isolithocholic acid | 0.641 | 4.70E-06 |
|  | Bacteroidetes | *Alistipes* | Fatty acids | Methylglutaric acid | 0.63 | 7.80E-06 |
|  | Firmicutes | *Enterococcus* | Amino acids | L-Norleucine | 0.626 | 9.30E-06 |
|  | Firmicutes | *Enterococcus* | Amino acids | L-Leucine | 0.622 | 1.10E-05 |
|  | Firmicutes | *Fusicatenibacter* | Benzenoids | Phthalic acid | 0.646 | 3.80E-06 |
|  | Firmicutes | *Gemmiger* | Benzenoids | Phthalic acid | 0.602 | 2.50E-05 |
|  | Firmicutes | *Ruminococcus* | Benzenoids | Phthalic acid | 0.6 | 2.70E-05 |
|  | Firmicutes | *Clostridium IV* | Bile acids | isolithocholic acid | 0.73 | 4.10E-08 |
|  | Firmicutes | *Clostridium IV* | Bile acids | Lithocholic acid | 0.682 | 6.40E-07 |
|  | Firmicutes | *Romboutsia* | Bile acids | Hyodeoxycholic acid | 0.665 | 1.50E-06 |
|  | Firmicutes | *Clostridium IV* | Bile acids | Hyodeoxycholic acid | 0.633 | 6.70E-06 |
|  | Firmicutes | *Blautia* | Bile acids | Hyodeoxycholic acid | 0.631 | 7.40E-06 |
|  | Firmicutes | *Romboutsia* | Fatty acids | Valeric acid | 0.713 | 1.10E-07 |
|  | Firmicutes | *Clostridium IV* | Fatty acids | Methylglutaric acid | 0.701 | 2.30E-07 |
|  | Firmicutes | *Roseburia* | Fatty acids | Valeric acid | 0.681 | 6.90E-07 |
|  | Firmicutes | *Gemmiger* | Fatty acids | Valeric acid | 0.679 | 7.80E-07 |
|  | Firmicutes | *Clostridium IV* | Fatty acids | 3-Methyladipic acid | 0.65 | 3.10E-06 |
|  | Firmicutes | *Blautia* | Fatty acids | Valeric acid | 0.646 | 3.80E-06 |
|  | Firmicutes | *Fusicatenibacter* | Fatty acids | Valeric acid | 0.644 | 4.30E-06 |
|  | Firmicutes | *Coprococcus* | Fatty acids | Valeric acid | 0.64 | 4.90E-06 |
|  | Firmicutes | *Faecalibacterium* | Fatty acids | Valeric acid | 0.638 | 5.50E-06 |
|  | Firmicutes | *Clostridium IV* | Fatty acids | Azelaic acid | 0.625 | 9.80E-06 |
|  | Firmicutes | *Clostridium IV* | Fatty acids | Pimelic acid | 0.622 | 1.10E-05 |
|  | Firmicutes | *Romboutsia* | Fatty acids | Caproic acid | 0.608 | 1.90E-05 |
|  | Firmicutes | *Gemmiger* | Fatty acids | 3-Methyladipic acid | 0.606 | 2.10E-05 |
|  | Firmicutes | *Clostridium IV* | Fatty acids | Valeric acid | 0.605 | 2.20E-05 |
|  | Firmicutes | *Ruminococcus2* | Fatty acids | Valeric acid | 0.603 | 2.40E-05 |
|  | Firmicutes | *Clostridium XlVa* | Organic acids | Itaconic acid | 0.623 | 1.10E-05 |
|  | Proteobacteria | *Proteus* | Amino acids | L-Arginine | 0.609 | 1.80E-05 |
|  | Proteobacteria | *Parasutterella* | Fatty acids | 3_Methyladipic acid | 0.612 | 1.70E-05 |
| **Negative correlations** | Actinobacteria | *Adlercreutzia* | Fatty acids | 2-Hydroxy-3-methylbutyric acid | -0.634 | 6.70E-06 |
|  | Actinobacteria | *Actinomyces* | Fatty acids | Butyric acid | -0.606 | 2.10E-05 |
|  | Bacteroidetes | *Alistipes* | Bile acids | Glycocholic acid | -0.628 | 8.50E-06 |
|  | Bacteroidetes | *Alistipes* | Bile acids | Taurocholic acid | -0.628 | 8.60E-06 |
|  | Firmicutes | *Roseburia* | Amino acids | L-Valine | -0.696 | 3.10E-07 |
|  | Firmicutes | *Clostridium IV* | Amino acids | L-Valine | -0.691 | 4.20E-07 |
|  | Firmicutes | *Roseburia* | Amino acids | L-Leucine | -0.687 | 5.00E-07 |
|  | Firmicutes | *Ruminococcus* | Amino acids | L-Valine | -0.682 | 6.60E-07 |
|  | Firmicutes | *Romboutsia* | Amino acids | L-Cystine | -0.672 | 1.10E-06 |
|  | Firmicutes | *Roseburia* | Amino acids | L-Norleucine | -0.669 | 1.30E-06 |
|  | Firmicutes | *Ruminococcus* | Amino acids | L-Cystine | -0.662 | 1.80E-06 |
|  | Firmicutes | *Clostridium IV* | Amino acids | L-Cystine | -0.658 | 2.20E-06 |
|  | Firmicutes | *Romboutsia* | Amino acids | L-Valine | -0.645 | 4.00E-06 |
|  | Firmicutes | *Clostridium XlVb* | Amino acids | L-Cystine | -0.641 | 4.80E-06 |
|  | Firmicutes | *Ruminococcus* | Amino acids | L-Leucine | -0.639 | 5.30E-06 |
|  | Firmicutes | *Roseburia* | Amino acids | L-Cystine | -0.625 | 9.70E-06 |
|  | Firmicutes | *Gemmiger* | Amino acids | L-Arginine | -0.622 | 1.10E-05 |
|  | Firmicutes | *Ruminococcus* | Amino acids | L-Norleucine | -0.617 | 1.40E-05 |
|  | Firmicutes | *Clostridium IV* | Amino acids | L-Leucine | -0.616 | 1.40E-05 |
|  | Firmicutes | *Roseburia* | Amino acids | L-Phenylalanine | -0.616 | 1.40E-05 |
|  | Firmicutes | *Ruminococcus* | Amino acids | L-Tryptophan | -0.612 | 1.70E-05 |
|  | Firmicutes | *Faecalibacterium* | Amino acids | L-Leucine | -0.611 | 1.70E-05 |
|  | Firmicutes | *Clostridium IV* | Amino acids | L-Histidine | -0.608 | 1.90E-05 |
|  | Firmicutes | *Gemmiger* | Amino acids | L-Leucine | -0.603 | 2.40E-05 |
|  | Firmicutes | *Faecalibacterium* | Amino acids | L-Norleucine | -0.601 | 2.60E-05 |
|  | Firmicutes | *Clostridium IV* | Bile acids | Taurocholic acid | -0.686 | 5.30E-07 |
|  | Firmicutes | *Granulicatella* | Bile acids | Lithocholic acid | -0.636 | 5.90E-06 |
|  | Firmicutes | *Clostridium IV* | Bile acids | Glycocholic acid | -0.634 | 6.60E-06 |
|  | Firmicutes | *Romboutsia* | Fatty acids | 2-Hydroxy-3-methylbutyric acid | -0.651 | 3.10E-06 |
|  | Firmicutes | *Fusicatenibacter* | Fatty acids | 2-Hydroxy-3-methylbutyric acid | -0.637 | 5.70E-06 |
|  | Firmicutes | *Roseburia* | Fatty acids | 2-Hydroxy-3-methylbutyric acid | -0.621 | 1.20E-05 |
|  | Firmicutes | *Ruminococcus* | Fatty acids | 2-Hydroxy-3-methylbutyric acid | -0.619 | 1.20E-05 |
|  | Firmicutes | *Clostridium IV* | Fatty acids | 2-Hydroxy-3-methylbutyric acid | -0.603 | 2.40E-05 |
|  | Firmicutes | *Coprococcus* | Fatty acids | 2-Hydroxy-3-methylbutyric acid | -0.6 | 2.70E-05 |
|  | Firmicutes | *Clostridium IV* | Organic acids | L-Lactic acid | -0.728 | 4.60E-08 |
|  | Firmicutes | *Clostridium IV* | Organic acids | 2-Hydroxybutyric acid | -0.672 | 1.10E-06 |
|  | Proteobacteria | *Proteus* | Bile acids | Hyodeoxycholic acid | -0.677 | 8.50E-07 |
|  | Proteobacteria | *Proteus* | Bile acids | Lithocholic acid | -0.644 | 4.20E-06 |
|  | Proteobacteria | *Proteus* | Bile acids | Deoxycholic acid | -0.635 | 6.20E-06 |
|  | Proteobacteria | *Proteus* | Fatty acids | Methylglutaric acid | -0.611 | 1.70E-05 |

**Table S5. Fungal genera correlated with the PCDAI.**

| **Genus** | **R** | **P** |  | **Positive** |
| --- | --- | --- | --- | --- |
|  | **(Spearman index)** |  | **FDR** | **Samples** |
| *Galactomyces* | -0.48 | 0.00329 | 0.18 | 26 |
| *Rhodosporidium* | 0.41 | 0.013458 | 0.71 | 17 |
| *Kabatiella* | 0.35 | 0.035006 | 1 | 31 |
